# Supplementary material for: PBRM1 directs PBAF to pericentromeres and protects centromere integrity
Source: Nat Commun. 2025 Feb 26;16:1980. doi: 10.1038/s41467-025-57277-9 (PMC11865495; doi:10.1038/s41467-025-57277-9)
Supplement: Supplementary file 1 — Supplementary Information [file 41467_2025_57277_MOESM1_ESM.pdf]

PBRM1 directs PBAF to pericentromeres and protects centromere integrity

Karen A. Lane[*], Alison Harrod[*], Lillian Wu, Theodoros I. Roumeliotis, Hugang Feng, Shane Foo, Katheryn A. G. Begg, Federica Schiavoni, Noa Amin, Frank T. Zenke, Alan A. Melcher, Jyoti S. Choudhary, and Jessica A. Downs

Supplementary information & figures

Figure S1

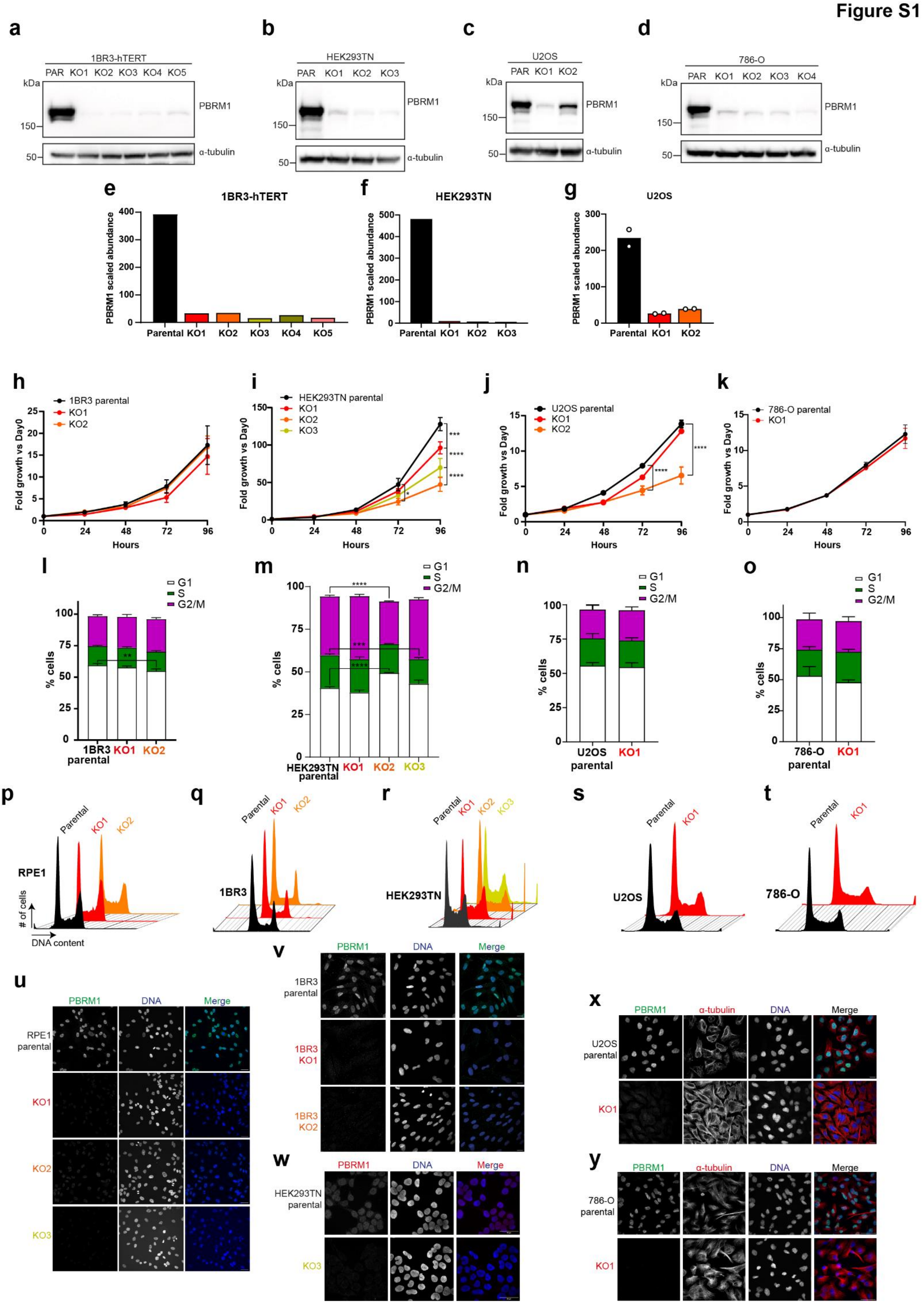
a
1BR3-hTERT
PAR KO1 KO2 KO3 KO4 KO5
kDa
PBRM1
α-tubulin
b
HEK293TN
PAR KO1 KO2 KO3
c
U2OS
PAR KO1 KO2
d
786-O
PAR KO1 KO2 KO3 KO4
e
1BR3-hTERT
PBRM1 scaled abundance
Parental KO1 KO2 KO3 KO4 KO5
f
HEK293TN
Parental KO1 KO2 KO3
g
U2OS
Parental KO1 KO2
h
1BR3 parental
KO1
KO2
Fold growth vs Day0
Hours
i
HEK293TN parental
KO1
KO2
KO3
j
U2OS parental
KO1
KO2
k
786-O parental
KO1
l
G1
S
G2/M
% cells
1BR3 parental KO1 KO2
m
HEK293TN parental KO1 KO2 KO3
n
U2OS parental KO1
o
786-O parental KO1
p
RPE1
Parental KO1 KO2
# of cells
DNA content
q
1BR3
Parental KO1 KO2
r
HEK293TN
Parental KO1 KO2 KO3
s
U2OS
Parental KO1
t
786-O
Parental KO1
u
PBRM1 DNA Merge
RPE1 parental
KO1
KO2
KO3
v
PBRM1 DNA Merge
1BR3 parental
1BR3 KO1
1BR3 KO2
w
PBRM1 DNA Merge
HEK293TN parental
KO3
x
PBRM1 α-tubulin DNA Merge
U2OS parental
KO1
y
PBRM1 α-tubulin DNA Merge
786-O parental
KO1

Supplementary Figure 1. Characterisation of PBRM1 KO cell lines. a-d Western blotting for PBRM1 in parental and PBRM1 knockouts of the indicated cell lines – (a) 1BR3-hTERT, (b) HEK293TN, (c) U2OS, and (d) 786-O. Note that one clone in U2OS (“HetKO2”) retains low levels of PBRM1 due to a heterozygous truncating mutation. α-tubulin was used as loading control. **e-g** Scaled abundances of PBRM1 in proteomic analyses of whole cell protein extracts of parental and PBRM1 knockouts in (e) 1BR3-hTERT, (f) HEK293TN, and (g) U2OS. Points in (g) correspond to two independent biological replicates. PBRM1 was not detected in proteomic analyses of 786-O cells. **h-k** Proliferation rates of PBRM1 knockouts in (h) 1BR3-hTERT (n=5), (i) HEK293TN (n=3, *p=0.0114, ***p=0.0004, ****p<0.0001), (j) U2OS (n=2 for KO1, n=4 for KO2, ****p<0.0001), and (k) 786-O (n=3), compared to parental cells. Cells were counted every 24 hours and fold change in cell number was calculated compared to the number of cells seeded. Data are presented as mean±SEM and were analysed by 2way ANOVA with Dunnett’s test. **l-o** Cell cycle distribution of parental and PBRM1 knockout cells in (l) 1BR3-hTERT (n=4, **p=0.0038), (m) HEK293TN (n=3, ***p=0.0005, ****p<0.0001), (n) U2OS (n=4), and (o) 786-O (n=4), measured using flow cytometry. Data are presented as mean±SEM and were analysed by 2way ANOVA with Dunnett’s test. **p-t** Representative histograms showing cell cycle profiles of parental and PBRM1 knockout (p) hTERT-RPE1, (q) 1BR3-hTERT, (r) HEK293TN, (s) U2OS, and (t) 786-O cells, corresponding to data shown in Figure 1(e) and Supplementary Figure 1(l)-(o). **u-y** Representative immunofluorescence images showing PBRM1 expression in parental and PBRM1 knockout cells in (u) hTERT-RPE1, (v) 1BR3-hTERT, (w) HEK293TN, (x) U2OS, and (y) 786-O, as well as nuclear (DNA, blue) staining. **x** & **y** also show cytoskeleton (α-tubulin, red) staining. Scale bars correspond to 20µm in **u**, **v**, & **x**, and correspond to 50 µm in **w** and **y**. Source data are provided as a source data file.

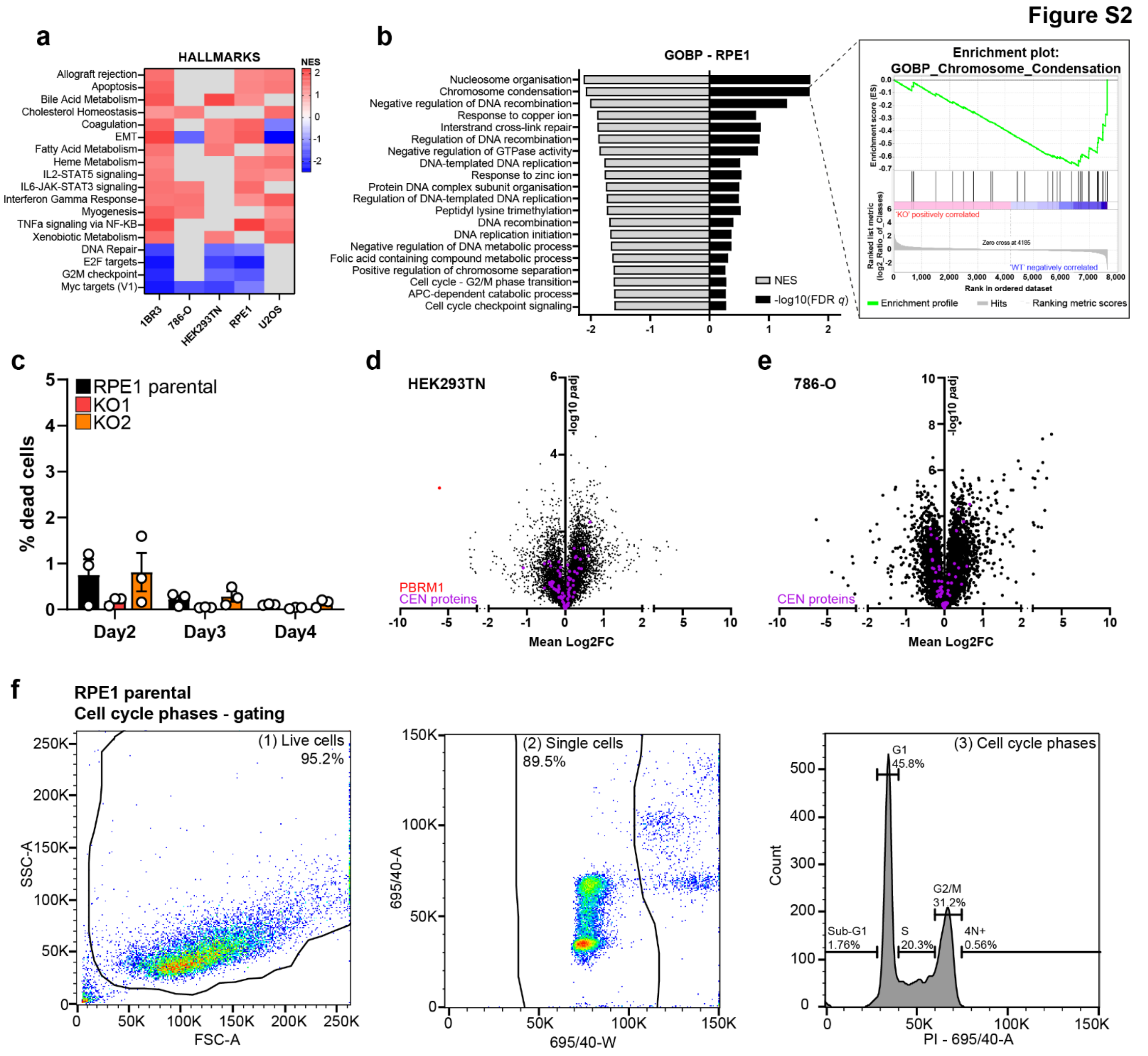

Supplementary Figure 2. Dysregulation of a number of pathways is conserved across PBRM1 KO cell lines. a Heatmap showing the normalised enrichment score (NES) of Hallmark gene sets associated with PBRM1 loss, determined using GSEA analysis. Red corresponds to positive enrichment in PBRM1 knockouts versus the corresponding parental cells, while blue corresponds to negatively enriched gene sets. Grey cells indicate gene sets which did not reach a significant enrichment level of FDR <0.25. Only Hallmark gene sets significantly enriched in 3 or more cell lines were included on heatmap. **b** GSEA gene ontology analysis of biological processes (GOBP) negatively enriched in RPE1 PBRM1 knockouts compared to parental cells. The 20 processes with the most significant FDR *q* values were included. NES (grey bars) of these enriched biological processes, as well as the -log10 FDR *q*-value (black bars) is shown. A representative enrichment plot of one of these biological processes, ‘Chromosome Condensation’, is shown on the right. **c** Quantification of the % of dead cells, defined as the % of propidium-iodide positive cells in a cell population, over the course of 4 days in culture, in RPE1 parental and PBRM1 knockouts. *n*=3, mean±SEM, with points indicating independent biological replicates, and data were analysed by 2way ANOVA with Dunnett’s test. **d-e** Protein abundances in PBRM1 knockouts compared to parental cells in (d) HEK293TN and (e) 786-O cell lines, detected using LC-MS of whole cell protein extracts. The mean Log2FC of protein abundance in PBRM1 knockouts versus parental cells is plotted against the -log10p. PBRM1 is highlighted in red, while peri/centromeric proteins are highlighted in purple. **f** Representative gating strategy for cell cycle analysis using flow cytometry in Figure 1 and Supplementary Figure 1. Cells were gated for live cells (left), single cells (centre), and remaining PI-stained cells were profiled for DNA content and assigned to the indicated cell cycle stages (right). Source data are provided as a source data file.

Figure S3

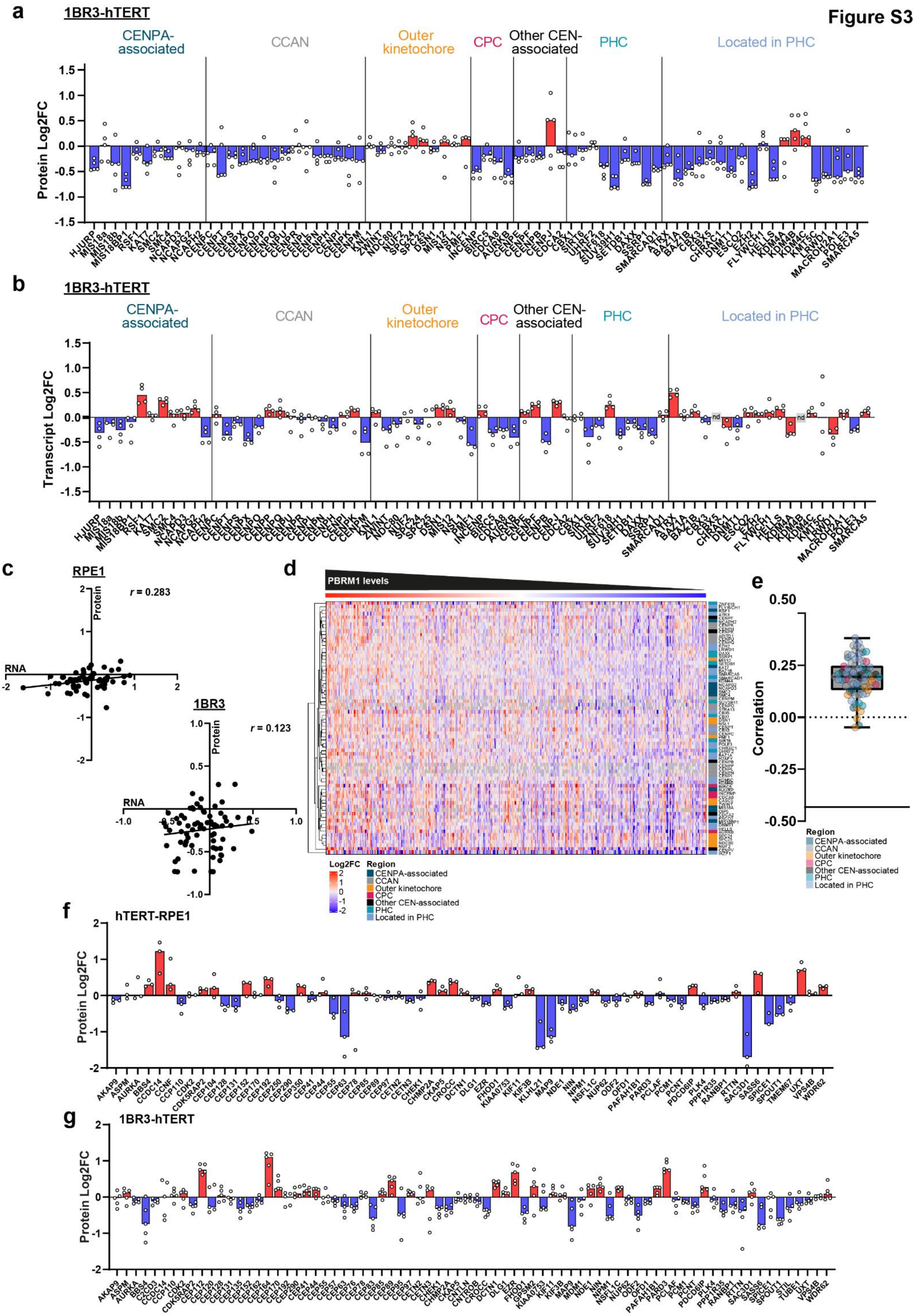
a
1BR3-hTERT
CENPA-associated
CCAN
Outer kinetochore
CPC
Other CEN-associated
PHC
Located in PHC
Protein Log2FC
b
1BR3-hTERT
Transcript Log2FC
nd
c
RPE1
r = 0.283
Protein
RNA
1BR3
r = 0.123
d
PBRM1 levels
Log2FC
Region
CENPA-associated
CCAN
Outer kinetochore
CPC
Other CEN-associated
PHC
Located in PHC
e
Correlation
Region
CENPA-associated
CCAN
Outer kinetochore
CPC
Other CEN-associated
PHC
Located in PHC
f
hTERT-RPE1
Protein Log2FC
g
1BR3-hTERT
Protein Log2FC

Supplementary Figure 3. PBRM1 KO cells show a loss of centromere proteins. a Log2FC of annotated centromere- and pericentromere-associated proteins in 1BR3 PBRM1 knockouts compared to parental cells. Points correspond to independent knockout clones. **b** Transcript levels of annotated centromere- & pericentromere-associated genes corresponding to the proteins in **a** were detected using RNA-seq. Median Log2FC of annotated genes transcribing centromere- & pericentromere-associated proteins in 1BR3 PBRM1 knockouts was plotted compared to parental cells. Points correspond to two individual knockout clones (KO1 and KO2) and two independent biological replicates. **c** Correlation plot showing Pearson correlation between protein and RNA levels of centromere & pericentromere protein/gene Log2FCs, in RPE1 (left) and 1BR3 (right) PBRM1 KOs compared to parental cells. Line indicates simple linear regression and Pearson correlation coefficient (*r*) is displayed. **d** Log2 fold changes of the indicated peri/centromere proteins in cancer cell lines from the CCLE database. Heatmap is ordered from highest to lowest PBRM1 Log2FC, left to right. **e** Pearson correlation of abundances of each protein in **d** versus PBRM1 abundance in cell lines from the CCLE database. Boxes contain the 25th to 75th percentiles with a line at median, and whiskers extend to the 10th and 90th percentiles, and individual points represent each protein from the indicated centromeric & pericentromeric regions. **f-g** Log2FC of annotated centrosome component and associated proteins in PBRM1 knockouts in (f) RPE1 and (g) 1BR3 cells compared to the parental of each cell line. Individual points represent independent PBRM1 knockout clones. Source data are provided as a source data file.

Figure S4

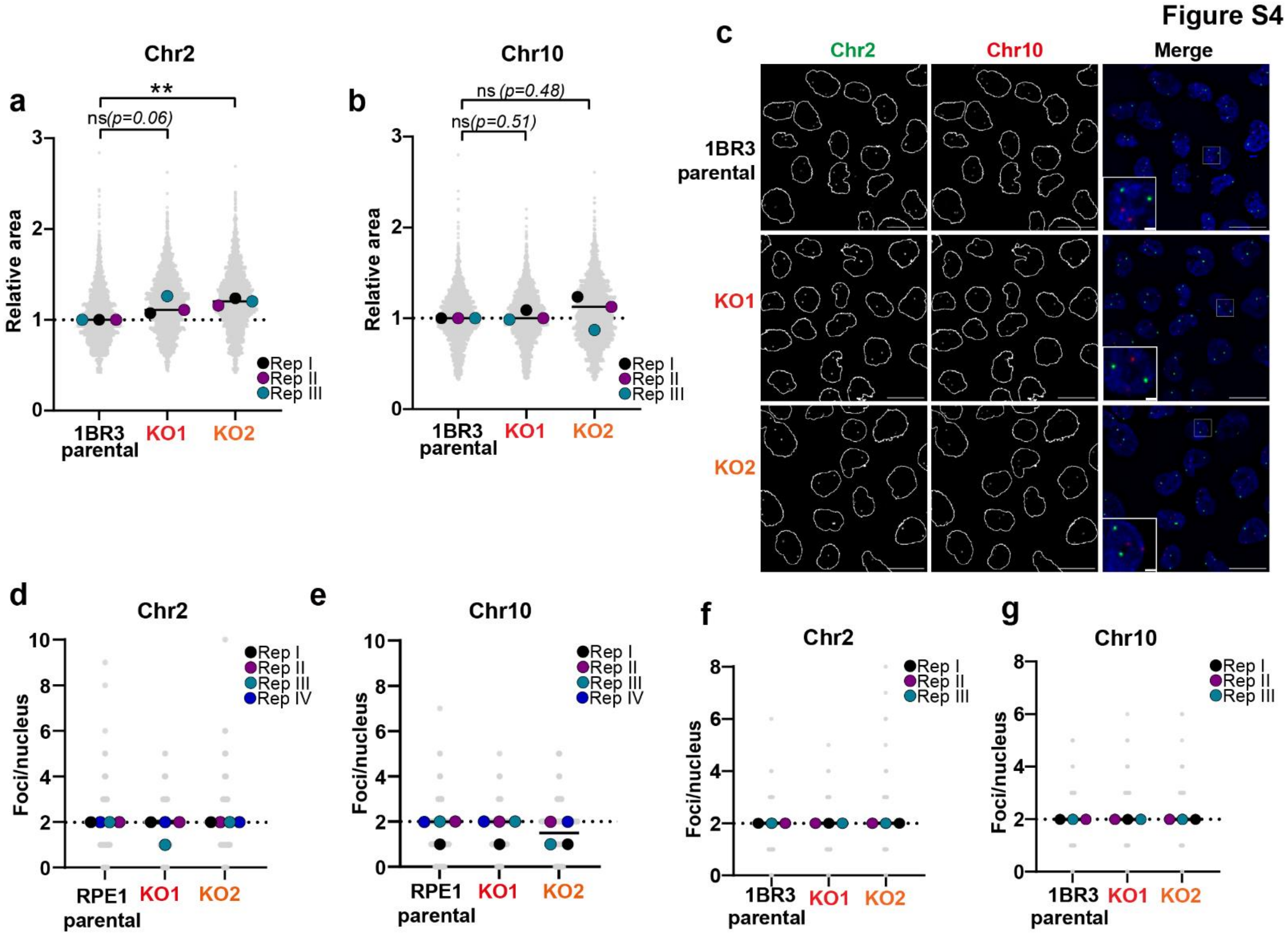

Supplementary Figure 4. PBRM1 knockouts have mildly increased α-satellite area. a-b Quantification of the area of individual foci in 1BR3 parental or PBRM1 knockout cells stained for α-satellite centromeric regions in (a) chromosome 2 and (b) chromosome 10, using FISH probes against α-satellite sequences in the corresponding chromosomes. *n*=3, grey points indicate individual foci, and coloured points represent the median of independent biological replicates, with line at the median of these replicates. Data were normalised to median area of parental cells and significance was determined using a two-sided t-test, ***p*=0.0061. **c** Representative images of α-satellite FISH of chromosomes 2 and 10 in 1BR3 parental and PBRM1 knockouts. Scale bars correspond to 20µm; in zoomed inset images, scale bars correspond to 5µm. **d-e** Quantification of the number of foci in RPE1 parental or PBRM1 knockout cells stained for α-satellite centromeric regions in (d) chromosome 2 and (e) chromosome 10, using FISH probes against α-satellite sequences in the corresponding chromosomes. *n*=3, grey points indicate individual foci, and coloured points represent the median of independent biological replicates, with line at the median of these replicates. **f-g** Quantification of the number of foci in 1BR3 parental or PBRM1 knockout cells stained for α-satellite centromeric regions in (f) chromosome 2 and (g) chromosome 10, using FISH probes against α-satellite sequences in the corresponding chromosomes. *n*=3, grey points indicate individual foci, and coloured points represent the median of independent biological replicates, with line at the median of these replicates. For RPE1, at least 1,000 foci were analysed per cell line. Source data are provided as a source data file.

Figure S5

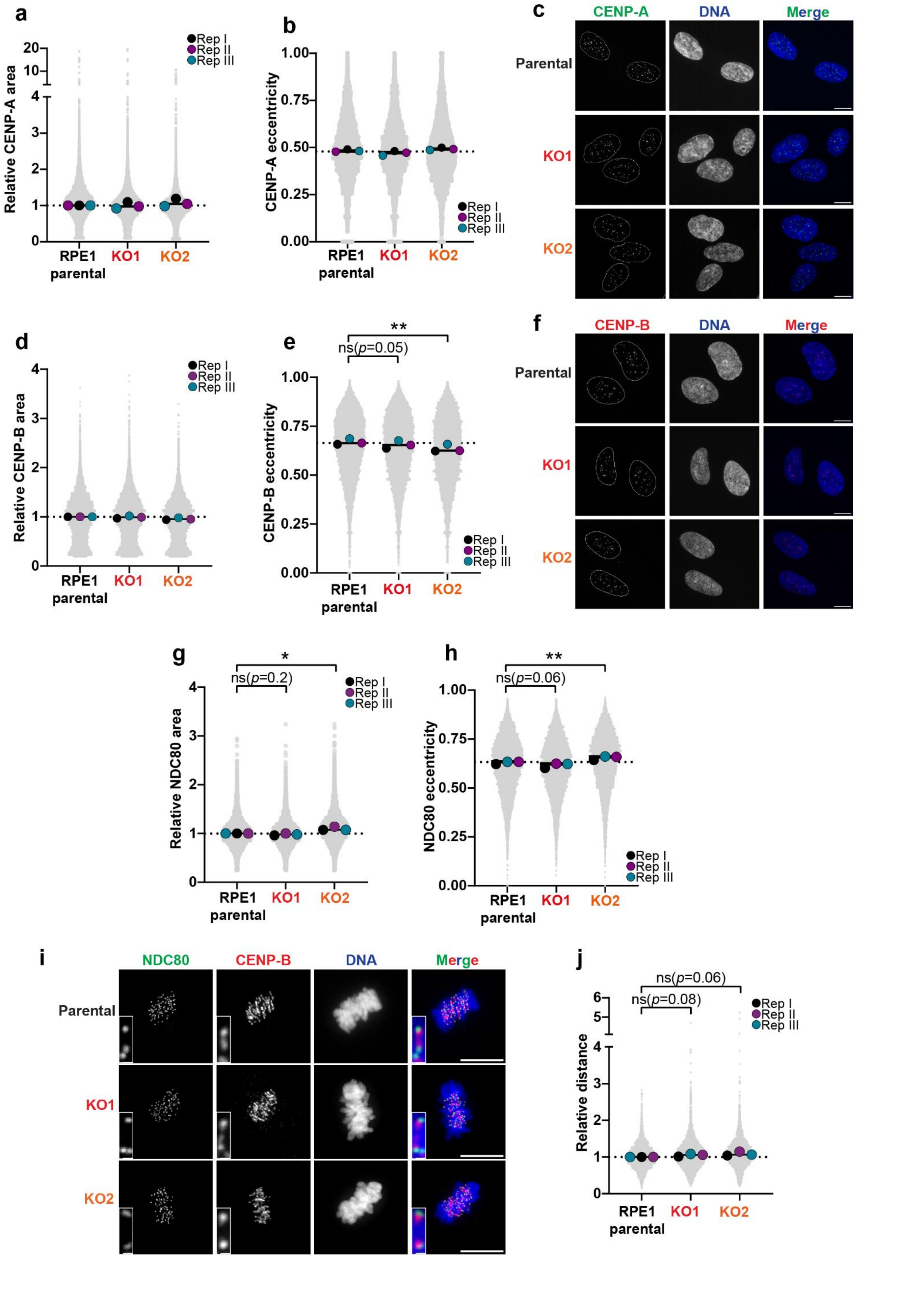
a
Relative CENP-A area
Rep I
Rep II
Rep III
RPE1 parental
KO1
KO2
b
CENP-A eccentricity
c
CENP-A
DNA
Merge
Parental
KO1
KO2
d
Relative CENP-B area
e
CENP-B eccentricity
ns(p=0.05)
**
f
CENP-B
g
Relative NDC80 area
ns(p=0.2)
*
h
NDC80 eccentricity
ns(p=0.06)
**
i
NDC80
j
Relative distance
ns(p=0.08)
ns(p=0.06)

Supplementary Figure 5. PBRM1 knockouts show altered structure of centromere-associated proteins. a-b Quantification of the (a) area and (b) shape (eccentricity) of CENP-A foci in RPE1 parental and PBRM1 KO cells. *n*=3, grey points indicate individual foci, and coloured points represent the median of independent biological replicates, with line at the median of these replicates. Data were normalised to median area of parental cells and differences were not significant based on a two-sided t-test. At least 300 cells were analysed for each condition. **c** Representative images showing CENP-A foci (green) in RPE1 parental and PBRM1 knockout cells. Scale bars correspond to 10µm. **d-e** Quantification of the (d) area and (e) shape (eccentricity) of CENP-B foci in RPE1 parental and PBRM1 KO cells. *n*=3, grey points indicate individual foci, and coloured points represent the median of independent biological replicates, with line at the median of these replicates. Data were normalised to median area of parental cells and were analysed using two-sided t-test, ***p*=0.0074. At least 150 cells were analysed for each condition. **f** Representative images showing CENP-B foci (red) in RPE1 parental and PBRM1 knockout cells. Scale bars correspond to 10µm. **g-h** Quantification of the (g) area and (h) shape (eccentricity) of NDC80 foci in RPE1 parental and PBRM1 KO cells in metaphase. *n*=3, grey points indicate individual foci, and coloured points represent the median of independent biological replicates, with line at the median of these replicates. Data were normalised to median area of parental cells and analysed using two-sided t-test, **p*=0.0486, ***p*=0.0058. At least 95 metaphases were analysed for each condition. **i** Representative images showing NDC80 (green) and CENP-B (red) in RPE1 parental and PBRM1 knockout cells. Scale bars correspond to 10µm. In zoomed images, scale bars correspond to 0.5µm. **j** Quantification of the distance between sister centromeres in dividing RPE1 parental and PBRM1 knockout cells, measured as the distance (µm) between NDC80 foci in metaphase nuclei. Sister kinetochores were identified via the connecting CENP-B signal between NDC80 foci. *n*=3, grey points indicate individual foci, and coloured points represent the median of independent biological replicates, with line at the median of these replicates. Data were normalised to median area of parental cells and analysed using two-sided t-test. Source data are provided as a source data file.

Figure S6

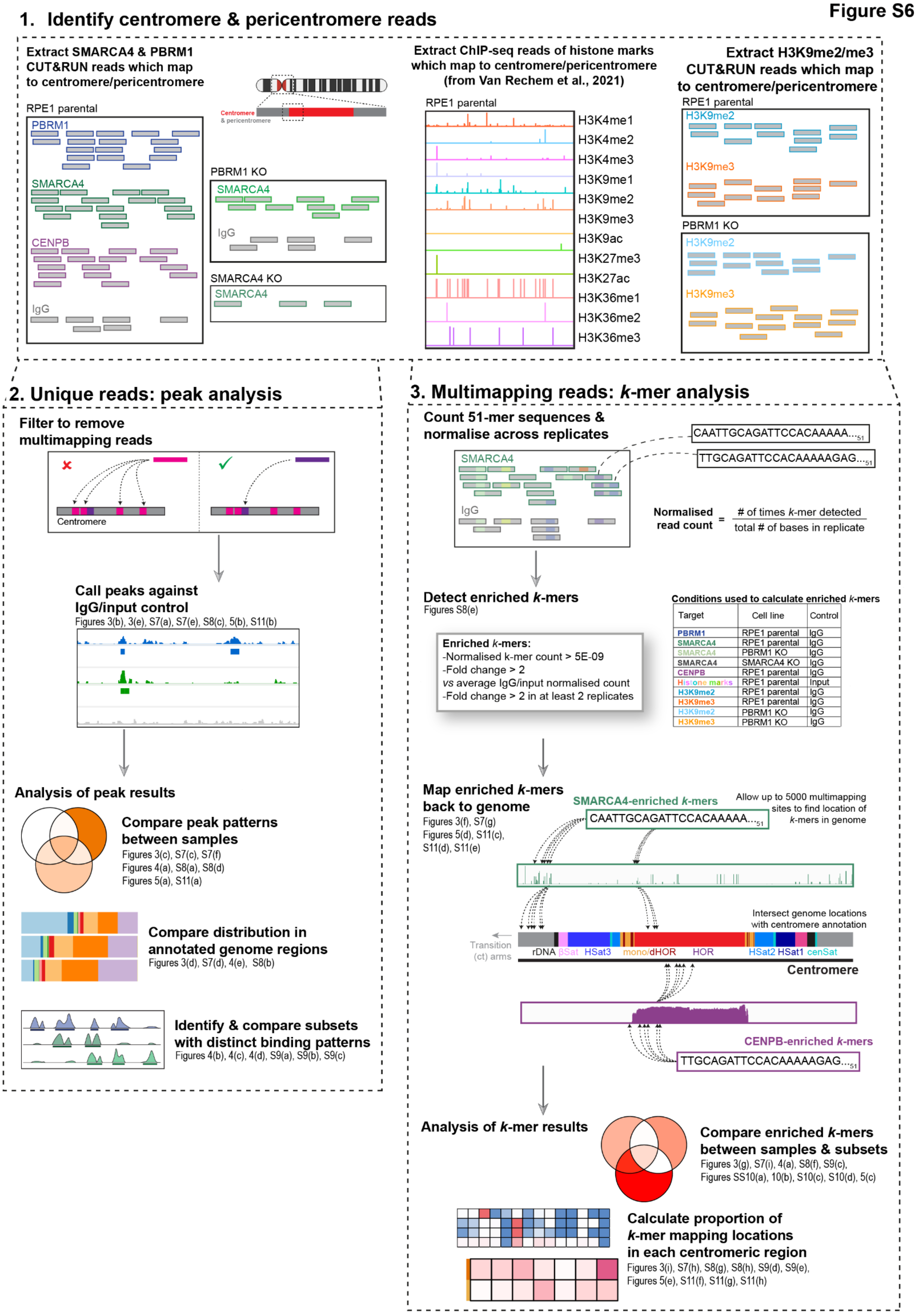

1. Identify centromere & pericentromere reads
Extract SMARCA4 & PBRM1 CUT&RUN reads which map to centromere/pericentromere
RPE1 parental
PBRM1
SMARCA4
CENPB
IgG
Centromere & pericentromere
PBRM1 KO
SMARCA4
IgG
SMARCA4 KO
SMARCA4
Extract ChIP-seq reads of histone marks which map to centromere/pericentromere (from Van Rechem et al., 2021)
RPE1 parental
H3K4me1
H3K4me2
H3K4me3
H3K9me1
H3K9me2
H3K9me3
H3K9ac
H3K27me3
H3K27ac
H3K36me1
H3K36me2
H3K36me3
Extract H3K9me2/me3 CUT&RUN reads which map to centromere/pericentromere
RPE1 parental
H3K9me2
H3K9me3
PBRM1 KO
H3K9me2
H3K9me3
2. Unique reads: peak analysis
Filter to remove multimapping reads
Centromere
Call peaks against IgG/input control
Figures 3(b), 3(e), S7(a), S7(e), S8(c), 5(b), S11(b)
Analysis of peak results
Compare peak patterns between samples
Figures 3(c), S7(c), S7(f)
Figures 4(a), S8(a), S8(d)
Figures 5(a), S11(a)
Compare distribution in annotated genome regions
Figures 3(d), S7(d), 4(e), S8(b)
Identify & compare subsets with distinct binding patterns
Figures 4(b), 4(c), 4(d), S9(a), S9(b), S9(c)
3. Multimapping reads: k-mer analysis
Count 51-mer sequences & normalise across replicates
CAATTGCAGATTCCACAAAAA...51
TTGCAGATTCCACAAAAAGAG...51
SMARCA4
IgG
Normalised read count = # of times k-mer detected / total # of bases in replicate
Detect enriched k-mers
Figures S8(e)
Enriched k-mers:
-Normalised k-mer count > 5E-09
-Fold change > 2
vs average IgG/input normalised count
-Fold change > 2 in at least 2 replicates
Conditions used to calculate enriched k-mers
Target | Cell line | Control
PBRM1 | RPE1 parental | IgG
SMARCA4 | RPE1 parental | IgG
SMARCA4 | PBRM1 KO | IgG
SMARCA4 | SMARCA4 KO | IgG
CENPB | RPE1 parental | IgG
Histone marks | RPE1 parental | Input
H3K9me2 | RPE1 parental | IgG
H3K9me3 | RPE1 parental | IgG
H3K9me2 | PBRM1 KO | IgG
H3K9me3 | PBRM1 KO | IgG
Map enriched k-mers back to genome
Figures 3(f), S7(g)
Figures 5(d), S11(c), S11(d), S11(e)
SMARCA4-enriched k-mers
Allow up to 5000 multimapping sites to find location of k-mers in genome
CAATTGCAGATTCCACAAAAA...51
Intersect genome locations with centromere annotation
Transition (ct) arms
rDNA
βSat
HSat3
mono/dHOR
HOR
HSat2
HSat1
cenSat
Centromere
CENPB-enriched k-mers
TTGCAGATTCCACAAAAAGAG...51
Analysis of k-mer results
Compare enriched k-mers between samples & subsets
Figures 3(g), S7(i), 4(a), S8(f), S9(c),
Figures SS10(a), 10(b), S10(c), S10(d), 5(c)
Calculate proportion of k-mer mapping locations in each centromere region
Figures 3(i), S7(h), S8(g), S8(h), S9(d), S9(e),
Figures 5(e), S11(f), S11(g), S11(h)

Supplementary Figure 6. Detailed workflow for the mapping strategy of CUT&RUN and ChIP-seq reads to centromeric and pericentromeric sequences. Workflow for mapping SWI/SNF subunits, CENP-B, and H3K9me2/3 CUT&RUN and publicly available ChIP-seq reads to centromeric and pericentromeric sequences (Box 1), either by calling peaks only on uniquely mapping reads (Box 2), or by taking all reads that mapped to the centromere and pericentromere, including multimapping reads at repetitive sequences, using *k*-mer enrichment analysis (Box 3). Detailed steps are described in Methods. Briefly, enriched peaks of uniquely mapping reads were compared between conditions and distribution of peaks among features was determined. For enriched *k*-mer analysis, sequencing reads which mapped to the centromeric and pericentromeric regions were extracted and 51-mer sequences in these reads were counted, normalised and compared across samples. Enriched *k*-mers were compared between conditions and were also mapped back to the genome and intersected with centromere annotation to determine where enriched *k*-mers are located. Data corresponding to these analyses are presented in Figures 3, 4 and 5, and Supplementary Figures 7-11, as indicated beside each stage of the analysis workflow.

Figure S7

a

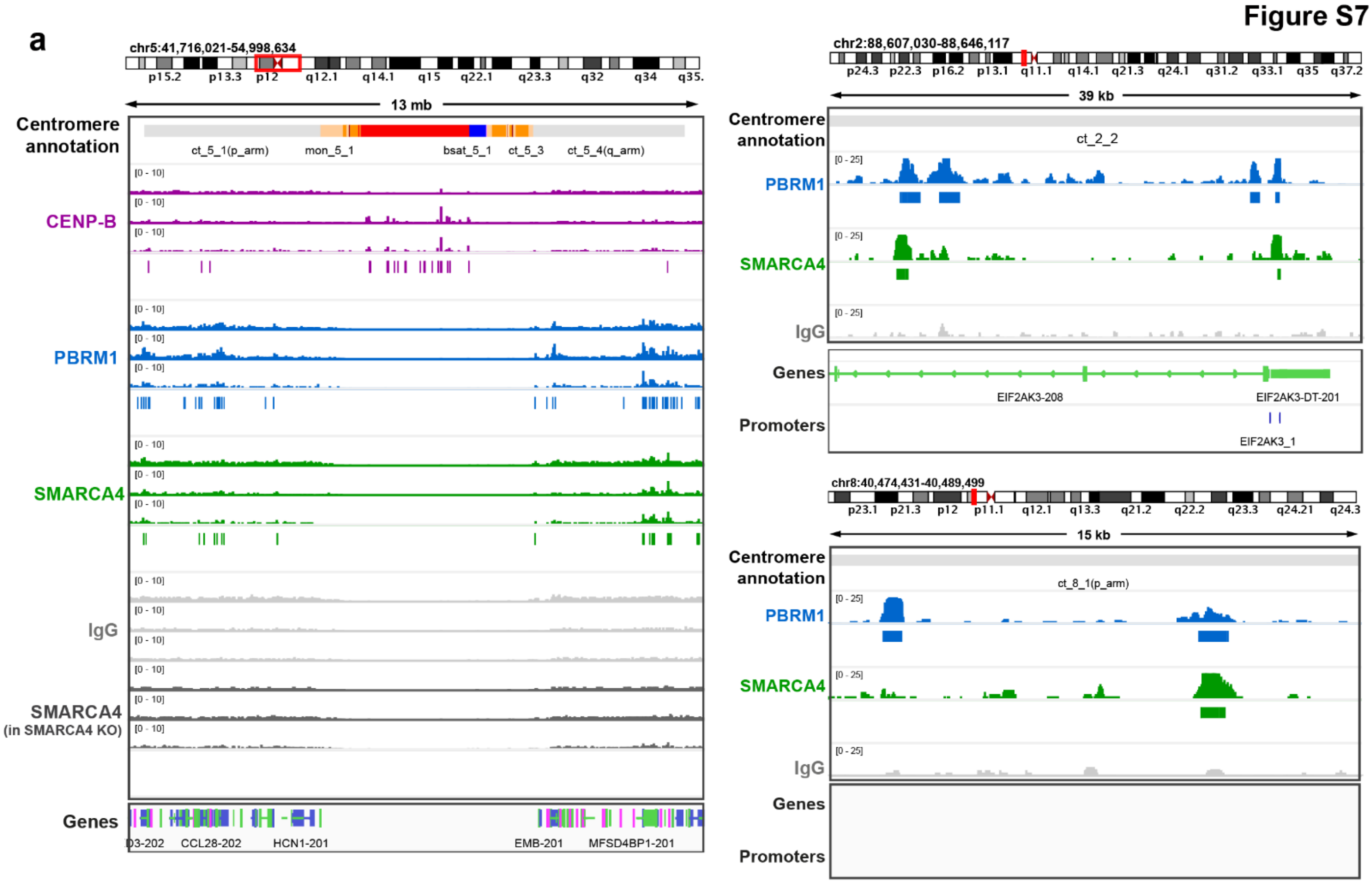

b

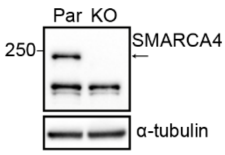

c

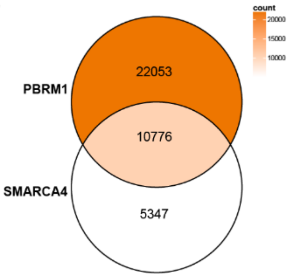

d

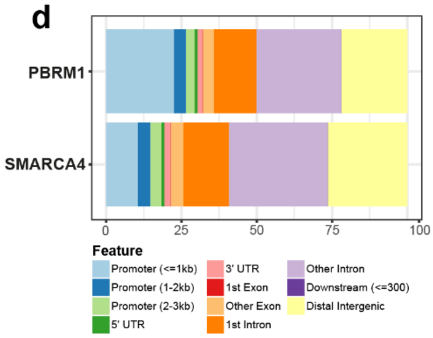

e

	Total peaks	
	CEN/ peri-CEN	Whole genome
PBRM1	1,181	32,706
SMARCA4	596	16,123
CENPB	511	1,957

f

	% overlap with			
	CEN/peri-CEN		Whole genome	
Sample	PBRM1	SMARCA4	PBRM1	SMARCA4
PBRM1		33.4		32.9
SMARCA4	66.1		66.8	

g

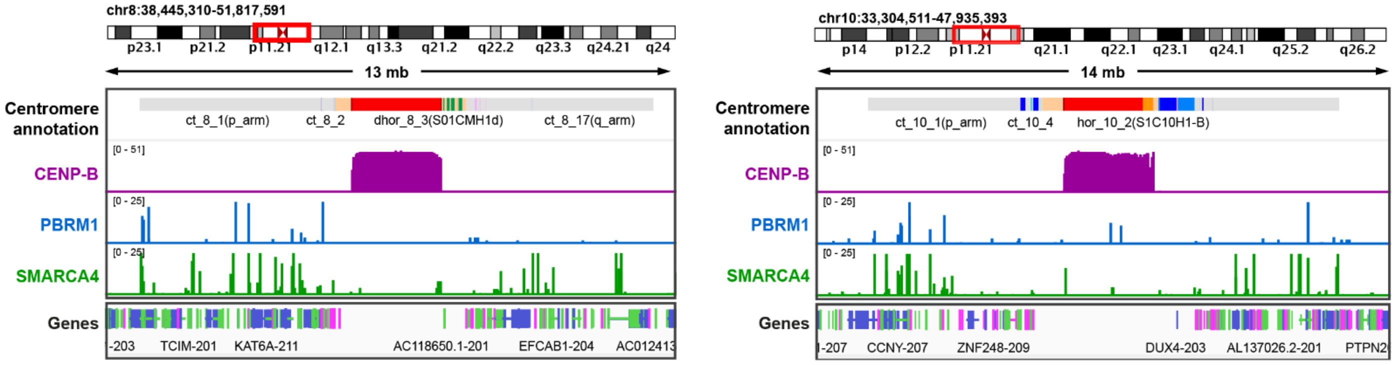

h

	βSat	censat	ct	dhor	gsat	hor	hsat1	hsat2	hsat3	hsat4	hsat5	mon	rDNA
CENPB	192	356	23429	21467	2	877112	4536	79	219	1	12	2535	1085
SMARCA4	490	469	156513	4	8	232	2275	76	138	0	0	92	0
PBRM1	58	411	85294	5	7	88	47	2	19	0	0	14	25
SMARCA4 KO	37	96	671	6	0	143	9697	16	63	1	0	91	317

i

	% overlap with	
	CEN/peri-CEN	
Sample	PBRM1	SMARCA4
PBRM1		32.0
SMARCA4	17.0	

Supplementary Figure 7. SWI/SNF binding in centromeric and pericentromeric regions. a Additional examples of representative genome tracks displaying coverage of reads from CENP-B (purple), PBRM1 (blue), SMARCA4 (green) and IgG control (grey) CUT&RUN sequencing in RPE1 parental cells, and SMARCA4 in SMARCA4 KO RPE1 cells as an additional control (dark grey). The full centromere is shown (left), and two examples of a zoomed in view of the centromere transition (ct) arms (right), excluding the CENP-B track. **b** Western blotting for SMARCA4 expression in whole cell lysates from RPE1 parental and SMARCA4 knockout cells used in CUT&RUN experiments. α-tubulin is used as loading control. **c** Venn diagram indicating the overlap of significantly enriched peaks in the whole genome, in SMARCA4 and PBRM1 in RPE1 parental cells versus their IgG control. Venn diagram shows enriched peaks found in at least two out of the three independent biological replicates, versus their IgG controls. The colour corresponds to the total number of enriched peaks in each region of the Venn diagram (count). **d** Stacked colour bar representing the genomic distribution of enriched PBRM1 and SMARCA4 peaks, categorised by feature, across the whole genome. **e** Table showing the total number of enriched peaks in PBRM1, SMARCA4, and CENP-B CUT&RUN sequencing in RPE1 parental cells, both in the centromere and pericentromere (‘CEN/peri-CEN’) and in the whole genome. **f** Table with percentage overlaps of peaks from the Venn diagrams in Figure 3c and Supplementary Figure 7c, showing shared peaks in the centromere/pericentromere and whole genome. **g** Additional representative genome tracks displaying mapping locations of enriched *k*-mers across the centromere and pericentromere from analysis of CENP-B (purple), PBRM1 (blue) and SMARCA4 (green) CUT&RUN sequencing in RPE1 parental cells). Genomic location is indicated at the top. Centromeric region is indicated above the tracks and transcript annotation is shown below the tracks. **h** Numbers of enriched *k*-mers which could map to the indicated region of the centromere and pericentromere in each dataset. **i** Table with percentage overlaps of *k*-mers from the Venn diagram in Figure 3g. Source data are provided as a source data file.

Figure S8

a

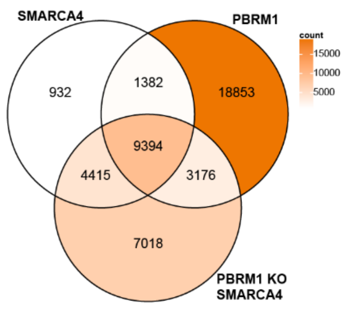

b

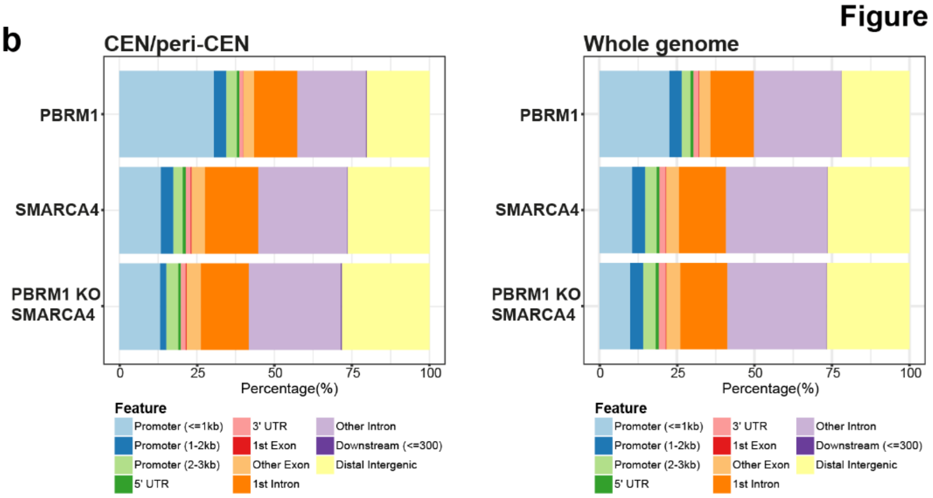

c

	Total peaks	
	CEN/ peri-CEN	Whole genome
PBRM1	1,181	32,706
SMARCA4	596	16,123
CENPB	511	1,957
PBRM1 KO SMARCA4	909	23,886

d

	% overlap with							
	CEN/peri-CEN				Whole genome			
	PBRM1	SMARCA4	PBRM1 KO SMARCA4	All 3	PBRM1	SMARCA4	PBRM1 KO SMARCA4	All 3
PBRM1		32.4	39.5	28.2		32.6	38.4	28.6
SMARCA4	66.1		85.1	56.9	66.8		85.6	58.3
PBRM1 KO SMARCA4	51.4	55.2		37.4	52.9	57.3		39.6

e

	Total *k*-mers
PBRM1	86,809
SMARCA4	163,292
CENPB	1,391,391
PBRM1 KO SMARCA4	218,466

f

	% overlap with			
	CEN/peri-CEN			
	PBRM1	SMARCA4	PBRM1 KO SMARCA4	All 3
PBRM1		32.0	30.7	25.5
SMARCA4	17.0		70.7	13.6
PBRM1 KO SMARCA4	12.2	52.8		10.1

g

	βSat	censat	ct	dhor	gsat	hor	hsat1	hsat2	hsat3	hsat4	hsat5	mon	rDNA
PBRM1	0.1	0.5	99.4	0.0	0.0	0.1	0.1	0.0	0.0	0.0	0.0	0.0	0.0
SMARCA4	0.3	0.3	97.8	0.0	0.0	0.1	1.4	0.0	0.1	0.0	0.0	0.1	0.0
PBRM1 KO SMARCA4	0.2	0.3	99.0	0.0	0.0	0.2	0.0	0.1	0.1	0.0	0.0	0.1	0.0

h

	βSat	censat	ct	dhor	gsat	hor	hsat1	hsat2	hsat3	hsat4	hsat5	mon	rDNA
PBRM1	52	358	58778	5	7	88	47	2	19	0	0	14	25
SMARCA4	6	53	26515	0	0	0	0	0	0	0	0	0	0
PBRM1 KO SMARCA4	142	363	94242	20	1	388	79	258	318	0	25	187	0

Supplementary Figure 8. SWI/SNF binding in PBRM1 KO cells is altered. a Venn diagram indicating the overlap of significantly enriched peaks across the whole genome, in SMARCA4 and PBRM1 in RPE1 parental cells (as per Supplementary Figure 7c), and SMARCA4 in PBRM1 KO cells. Venn diagrams shows enriched peaks found in at least two out of the three independent biological replicates, versus their IgG controls. The colour corresponds to the total number of enriched peaks in each region of the Venn diagram (count). **b** Stacked colour bars representing the genomic distribution of enriched PBRM1 and SMARCA4 peaks in RPE1 parental cells (as per Figure 3d and Supplementary Figure 7d) and SMARCA4 peaks in PBRM1 knockout cells, categorised by feature, in the centromere and pericentromere (left) and across the whole genome (right). **c** Table showing the total number of enriched PBRM1, SMARCA4, and CENP-B peaks in RPE1 parental cells (as per Supplementary Figure 7e), plus SMARCA4 peaks in PBRM1 KO cells, both in the centromere and pericentromere, and in the whole genome. **d** Table showing the % overlap of peaks called in PBRM1 and SMARCA4 CUT&RUN sequencing in RPE1 parental cells (as per Supplementary Figure 7f) as well as with SMARCA4 peaks enriched in PBRM1 KO cells, with “All 3” referring to the overlap of peaks in all 3 datasets. Overlapping peaks were calculated using bedtools intersect package, compared to the Intervene package for corresponding Venn diagrams in Figures 4a and Supplementary Figure 8a. **e** Table showing the total number of enriched PBRM1, SMARCA4, and CENP-B *k*-mers in RPE1 parental cells plus SMARCA4 in PBRM1 KO cells, in the centromere and pericentromere. **f** Table showing the % overlap of enriched *k*-mers in PBRM1 and SMARCA4 CUT&RUN sequencing in RPE1 parental cells (as per Supplementary Figure 7i) as well as with enriched *k*-mers in SMARCA4 in PBRM1 KO cells, with “All 3” referring to the overlap of peaks in all 3 datasets. Overlapping peaks were calculated using bedtools intersect package, compared to the Intervene package for the corresponding Venn diagram in Figure 4a. **g-h** Tables displaying (g) percentages and (h) numbers of enriched *k*-mers which could map to the indicated region of the centromere and pericentromere in each condition – PBRM1 and SMARCA4 in RPE1 parental cells (as per Figure 3i and Supplementary Figure 7h), as well as SMARCA4 in RPE1 PBRM1 knockout cells. Source data are provided as a source data file.

Figure S9

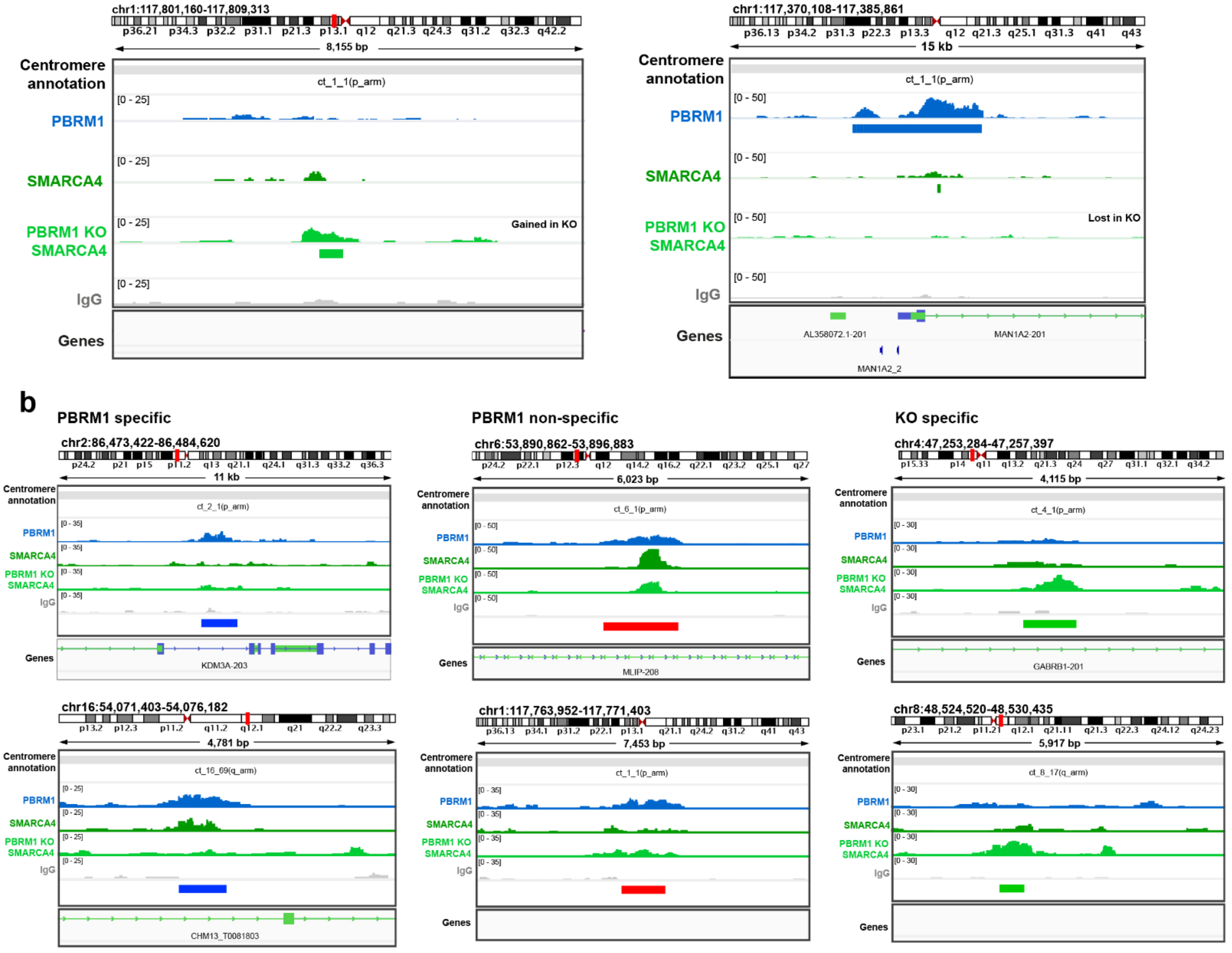

c

	Peaks	*k*-mers
**PBRM1 specific**	1,181	32,706
**PBRM1 non-specific**	596	16,123
**KO specific**	511	1,957

d

	βSat	censat	ct	dhor	gsat	hor	hsat1	hsat2	hsat3	hsat4	hsat5	mon	rDNA
**PBRM1 specific**	0.09	0.60	99.21	0.01	0.01	0.15	0.08	0.00	0.03	0.00	0.00	0.02	0.04
**PBRM1 non-specific**	0.02	0.20	99.97	0.00	0.00	0.00	0.00	0.00	0.00	0.00	0.00	0.00	0.00
**KO specific**	0.15	0.38	98.40	0.02	0.00	0.41	0.08	0.27	0.33	0.00	0.03	0.20	0.00

e

	βSat	censat	ct	dhor	gsat	hor	hsat1	hsat2	hsat3	hsat4	hsat5	mon	rDNA
**PBRM1 specific**	52	358	58778	5	7	88	47	2	19	0	0	14	25
**PBRM1 non-specific**	6	53	26515	0	0	0	0	0	0	0	0	0	0
**KO specific**	142	363	94242	20	1	388	79	258	318	0	25	187	0

Supplementary Figure 9. SWI/SNF binding pattern in the centromere and pericentromere changes in the absence of PBRM1 at sites of enriched histone marks in parental RPE1 cells. a Additional representative genome tracks displaying coverage of reads from PBRM1 (blue), SMARCA4 (green) and IgG control (grey) CUT&RUN sequencing in RPE1 parental cells and SMARCA4 in PBRM1 knockout cells (light green), showing an example of peaks gained (left) or lost (right) in PBRM1 knockout cells. One representative independent biological replicate is shown, with boxes underneath representing peaks that were called as significantly enriched (q<0.01) in at least two out of the three replicates versus their IgG control. **b** Representative genome tracks displaying coverage of reads from PBRM1 (blue), SMARCA4 (green) and IgG control (grey) CUT&RUN sequencing in RPE1 parental cells and SMARCA4 in PBRM1 knockout cells (light green) that highlight the three subsets of peaks (identified as shown in Figure 4c), represented as boxes below genome tracks: PBRM1 specific (blue, left), PBRM1 non-specific (red, middle) and KO specific (green, right). For genome browser tracks (a, b), genomic location is indicated at the top, centromeric annotation is shown above the tracks and transcript annotation is shown below. **c** Table showing the total number of enriched peaks and *k*-mers from the three subsets of peaks/*k*-mers (PBRM1 specific, PBRM1 non-specific, and KO specific), in the centromere and pericentromere. Subsets of *k*-mers were identified in the same way as peaks (shown in Figure 4c). **d-e** Tables displaying (d) percentages and (e) numbers of enriched *k*-mers which could map to the indicated region of the centromere and pericentromere in each subset of *k*-mers; PBRM1 specific, PBRM1 non-specific and KO-specific.

Figure S10

a

% population overlap w/histone marks

**Peaks**	H3K4me1	H3K4me2	H3K4me3	H3K9ac	H3K9me1	H3K9me2	H3K9me3	H3K27ac	H3K27me3	H3K36me1	H3K36me2	H3K36me3
**PBRM1 specific**	64.2	75.9	49.8	56.9	1.8	0.0	1.1	64.6	3.2	29.2	4.9	4.8
**PBRM1 non-specific**	95.1	96.1	43.6	70.2	4.3	0.0	0.9	90.3	0.2	29.2	9.7	2.8
**KO specific**	83.9	78.6	20.0	32.9	1.8	0.0	0.4	65.7	0.4	12.5	7.5	10.4

***k*-mers**	H3K4me1	H3K4me2	H3K4me3	H3K9ac	H3K9me1	H3K9me2	H3K9me3	H3K27ac	H3K27me3	H3K36me1	H3K36me2	H3K36me3
**PBRM1 specific**	0.0	3.7	18.8	1.9	0.0	0.0	0.0	1.0	0.0	0.0	0.0	0.0
**PBRM1 non-specific**	0.0	3.4	12.4	0.8	0.0	0.0	0.0	7.1	0.0	0.0	0.0	0.0
**KO specific**	0.0	0.1	0.6	0.0	0.0	0.0	0.2	0.3	0.0	0.0	0.0	0.0

b

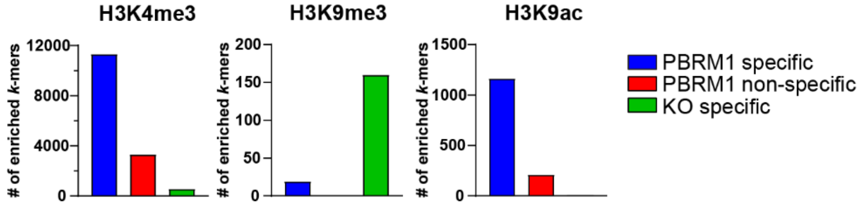

c

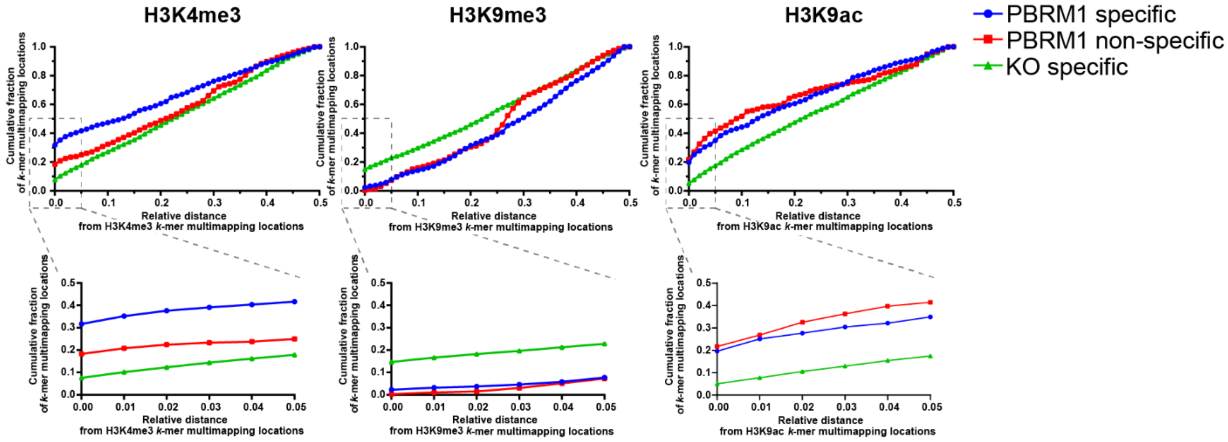

d

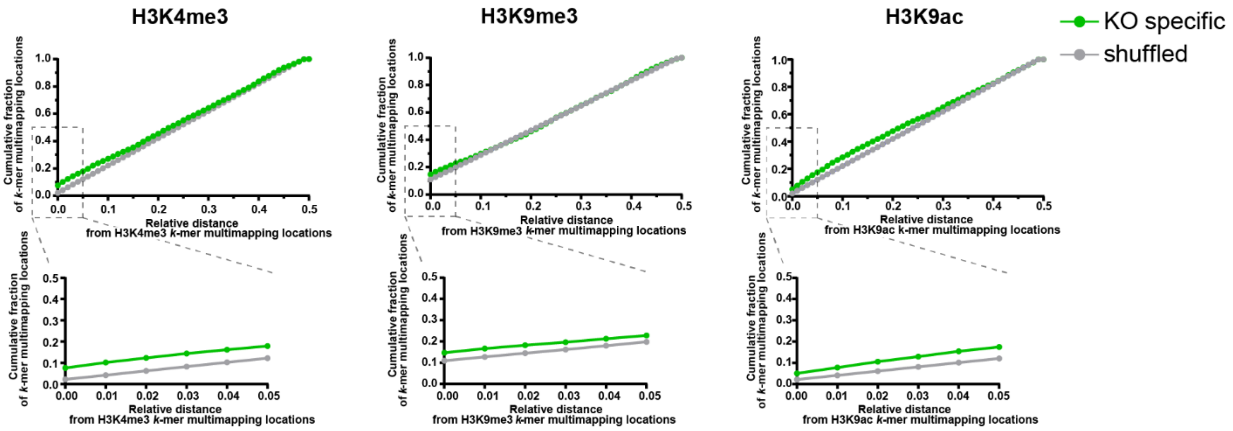

Supplementary Figure 10. PBAF association with histone modifications is altered in PBRM1 knockouts. a Tables containing the percentage overlap of each subset of peaks (top) or *k*-mers (bottom) from CUT&RUN sequencing data with enriched histone mark peaks or *k*-mers from RPE1 ChIP-seq data, specifically in centromeric and pericentromeric regions. A colour scale of low (white) to high (red) is shown on each table. **b** Graphs indicating the number of *k*-mers enriched in subsets (PBRM1 specific (blue), PBRM1 non-specific (red), and KO specific (green)) overlapping with enriched H3K4me3 (left), H3K9me3 (middle), or H3K9ac (right) *k*-mers. **c** Plots displaying the cumulative fraction of reference *k*-mer multimapping locations (PBRM1 specific (blue), PBRM1 non-specific (red) and KO specific (green)) at a relative distance from query histone mark *k*-mer multimapping locations (H3K4me3, left; H3K9me3, middle; H3K9ac, right). Relative distances are calculated as the shortest distance between each *k*-mer multimapping location in the query set and the two closest *k*-mer multimapping locations in the reference set, divided by the total distance between the two closest *k*-mer multimapping locations, so the maximum relative distance is 0.5. A zoomed in view of the smallest relative distances (0 to 0.05) is shown below each plot. **d** Plots displaying the cumulative fraction of reference *k*-mer multimapping locations (KO specific (green) and as a control (grey), shuffled locations of KO specific *k*-mer multimapping locations within the centromere and pericentromere at a relative distance from query histone mark *k*-mer multimapping locations (H3K4me3, left; H3K9me3, middle; H3K9ac, right). Relative distances are calculated as the shortest distance between each *k*-mer multimapping location in the query set and the two closest *k*-mer multimapping locations in the reference set, divided by the total distance between the two closest *k*-mer multimapping locations, so the maximum relative distance is 0.5. A zoomed in view of the smallest relative distances (0 to 0.05) is shown below each plot.

Figure S11

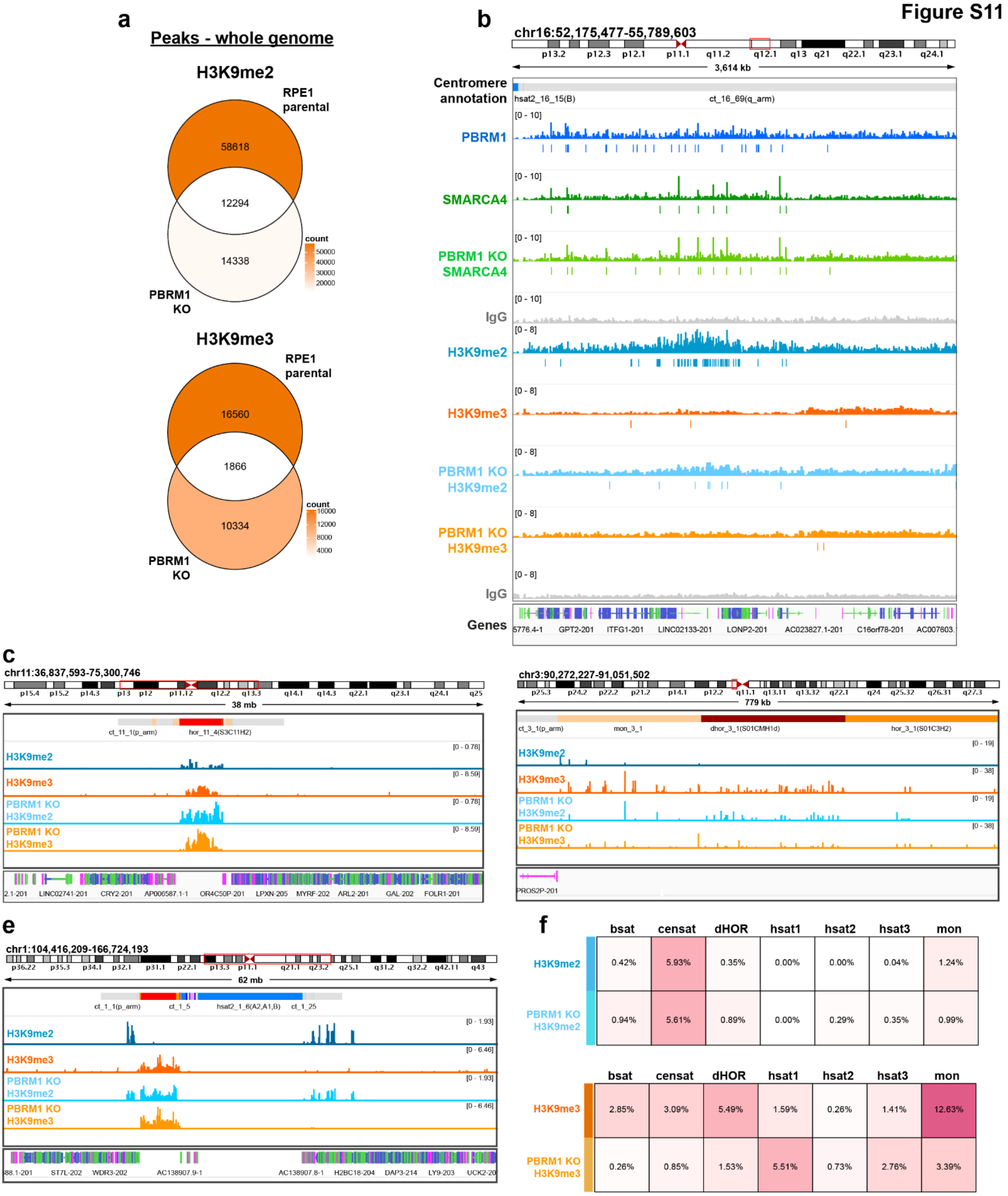

g

	βSat	censat	ct	dhor	gsat	hor	hsat1	hsat2	hsat3	hsat4	hsat5	mon	rDNA	tar
H3K9me2	0.42	5.93	86.30	0.35	0.00	7.62	0.00	0.00	0.04	0.03	0.87	1.24	0.00	0.00
PBRM1 KO H3K9me2	0.94	5.61	49.14	0.89	0.00	44.25	0.00	0.29	0.35	0.04	0.45	0.99	0.08	0.00
H3K9me3	2.85	3.09	40.28	5.49	0.00	57.87	1.59	0.26	1.41	0.96	0.00	12.63	0.24	0.00
PBRM1 KO H3K9me3	0.26	0.85	17.01	1.53	0.00	72.16	5.51	0.73	2.76	0.10	0.01	3.39	0.41	0.01

h

	βSat	censat	ct	dhor	gsat	hor	hsat1	hsat2	hsat3	hsat4	hsat5	mon	rDNA	tar
H3K9me2	149	2,108	30,689	125	0	2,710	0	0	15	11	310	442	1	0
PBRM1 KO H3K9me2	322	1,916	16,773	305	0	15,104	0	99	121	14	154	338	29	0
H3K9me3	1,209	1,312	17,112	2,333	0	24,581	675	111	601	407	1	5,364	101	0
PBRM1 KO H3K9me3	125	406	8,097	729	0	34,348	2,621	346	1,316	49	3	1,616	196	3

Supplementary Figure 11. H3K9 methylation pattern is altered in PBRM1-deficient cells. a Venn diagram indicating the overlap of significantly enriched peaks across the whole genome, in H3K9me2 (top) and H3K9me3 (bottom) in RPE1 parental cells and PBRM1 KO cells. Venn diagrams show enriched peaks found in at least two out of the three independent biological replicates, versus their IgG controls. The colour corresponds to the total number of enriched peaks in each region of the Venn diagram (count). **b** Additional representative genome tracks displaying coverage of reads from PBRM1 (blue), SMARCA4 (green), H3K9me2 (teal), H3K9me3 (orange) and IgG (grey) in RPE1 parental cells and SMARCA4 (light green), H3K9me2 (light blue) and H3K9me3 (light orange) in PBRM1 knockout cells. One representative independent biological replicate is shown, with boxes underneath representing peaks that were called as significantly enriched (q<0.01) in at least two replicates versus their IgG control. **c-e** Representative genome tracks displaying mapping locations of enriched *k*-mers across the centromere and pericentromere from analysis of H3K9me2 (teal) and H3K9me3 (orange) in RPE1 parental cells and PBRM1 knockout cells (light blue and light orange, respectively). Genome tracks show examples of altered *k*-mer enrichment in the HOR (c,e), the divergent HOR (dHOR) and monomeric repeats (mon) (d), and the transition arms (ct) (e) . **f** Percentage of enriched *k*-mers that map to indicated regions of centromere and pericentromere in parental and PBRM1 knockout RPE1 cells, plotted as a heatmap, for H3K9me2 (top) and H3K9me3 (bottom). **g-h** Tables displaying (g) percentages and (h) numbers of enriched *k*-mers which could map to the indicated region of the centromere and pericentromere in each condition – H3K9me2 and H3K9me3 in RPE1 parental cells and RPE1 PBRM1 knockout cells (as per Figure 5e and Supplementary Figure 11f).

Figure S12

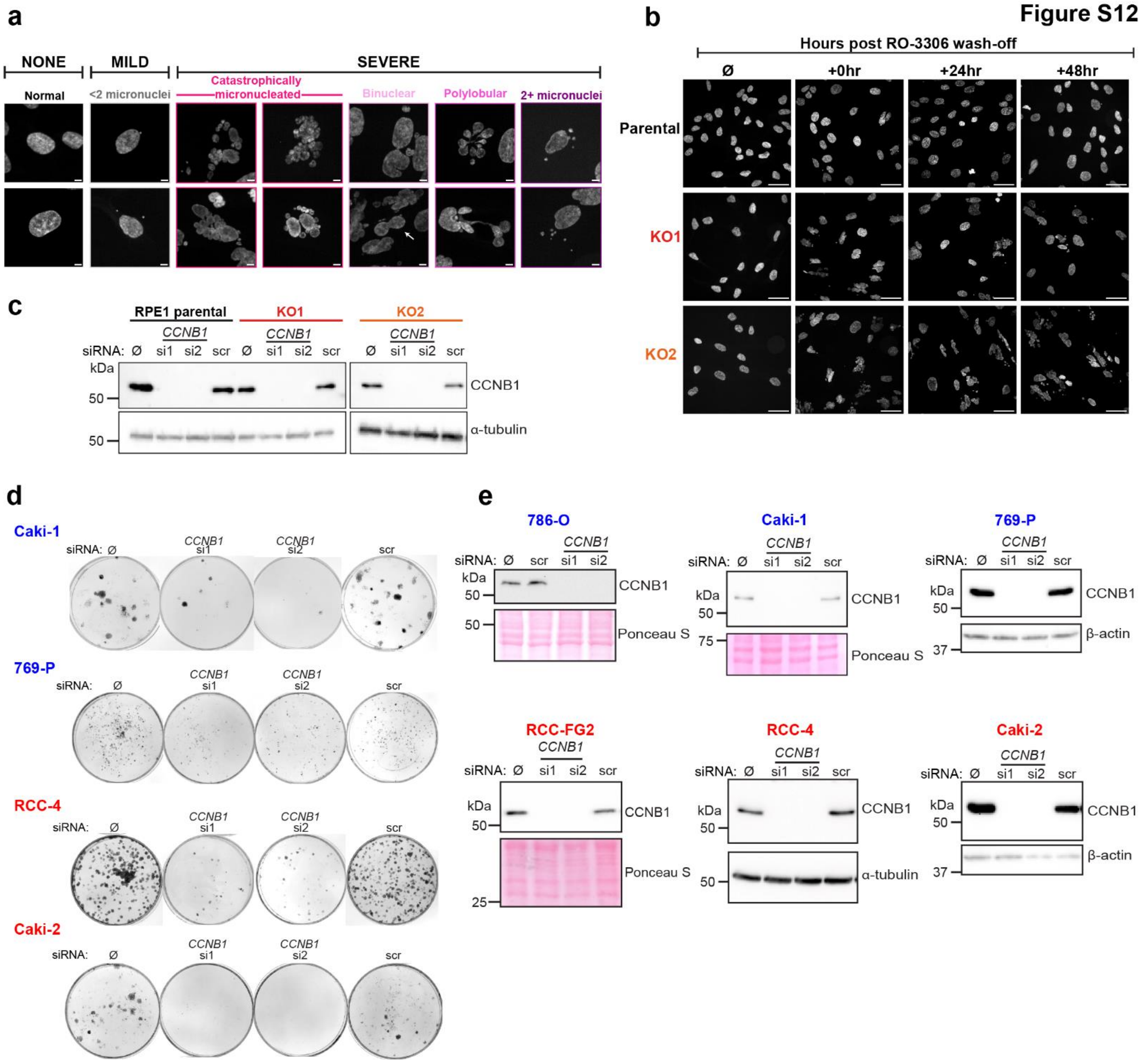

Supplementary Figure 12. PBRM1-deficient cells are sensitive to mitotic perturbation. a Representative images of nuclear defects and their associated morphological phenotypes, as quantified in Figure 6c-f. Scale bar represents 5µm. **b** Representative images from full timecourse in Figure 6f, where nuclear defects in RPE1 parental and PBRM1 knockout cells were quantified after treatment with 3µM RO-3306 for 24 hours (or treatment with DMSO alone (Ø)), and allowed to recover for 0hr, 24hr, or 48hr. Scale bars correspond to 40µm. **c** Representative western blot for CCNB1 following CCNB1 RNAi in RPE1 parental and PBRM1 knockout cells, corresponding to clonogenic survival assays in Figure 6g. **d** Representative images of colony formation following CCNB1 RNAi in a panel of PBRM1-proficient (blue) and -deficient (red) RCC cell lines, corresponding to data in Figure 6i. Representative images from 786-O and RCC-FG2 cell lines are shown in Figure 6k. **e** Representative western blots to detect CCNB1 protein expression after CCNB1 RNAi in a panel of PBRM1-proficient (blue) and -deficient (red) RCC cell lines, corresponding to data in Figure 6i. Source data are provided as a source data file.

Figure S13

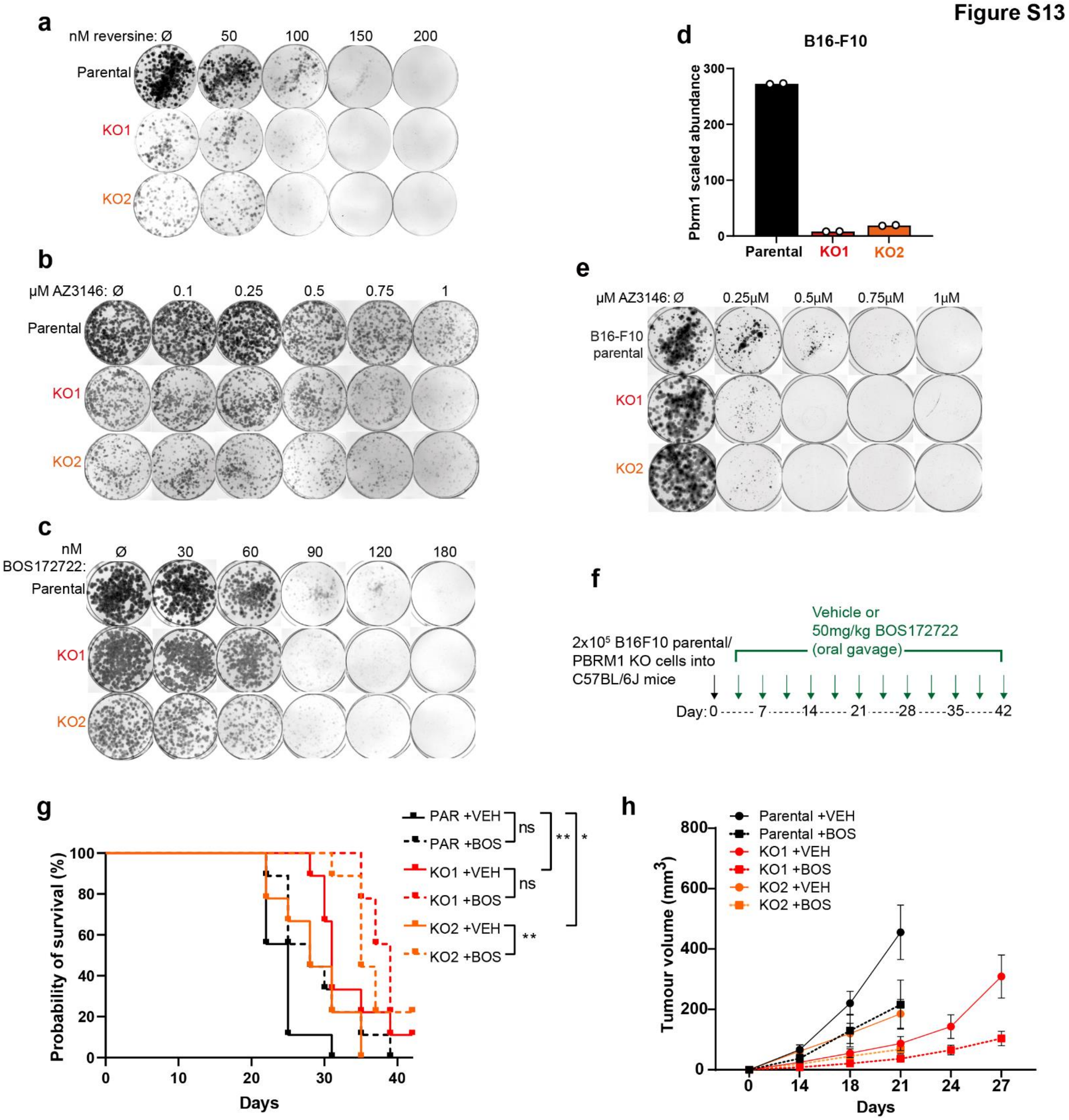
a
nM reversine: Ø 50 100 150 200
Parental
KO1
KO2
b
μM AZ3146: Ø 0.1 0.25 0.5 0.75 1
Parental
KO1
KO2
c
nM BOS172722: Ø 30 60 90 120 180
Parental
KO1
KO2
d
B16-F10
Pbrm1 scaled abundance
300
200
100
0
Parental KO1 KO2
e
μM AZ3146: Ø 0.25μM 0.5μM 0.75μM 1μM
B16-F10 parental
KO1
KO2
f
2x10⁵ B16F10 parental/ PBRM1 KO cells into C57BL/6J mice
Vehicle or 50mg/kg BOS172722 (oral gavage)
Day: 0 7 14 21 28 35 42
g
Probability of survival (%)
Days
PAR +VEH
PAR +BOS
KO1 +VEH
KO1 +BOS
KO2 +VEH
KO2 +BOS
ns
ns
**
**
*
h
Tumour volume (mm³)
Days
Parental +VEH
Parental +BOS
KO1 +VEH
KO1 +BOS
KO2 +VEH
KO2 +BOS

Supplementary Figure 13. PBRM1 knockouts are sensitive to Mps1 inhibition *in vitro* and *in vivo*. a-c Representative images of colony formation from clonogenic survival assays in Figure 7a-c, comparing survival of RPE1 parental and PBRM1 knockout cells to doses increasing doses of Mps1 inhibitors, compared to colony formation in DMSO-only treated cells (Ø). **d** Scaled abundances of Pbrm1 following LC-MS of whole cell protein extracts of B16-F10 parental and PBRM1 knockout cells. Points correspond to two independent biological replicates. **e** Representative images of colony formation from clonogenic survival assays in Figure 7f, comparing survival of B16-F10 parental and PBRM1 knockout cells treated with increasing doses of the Mps1 inhibitor AZ3146, compared to colony formation in DMSO-only treated cells (Ø). **f** Treatment outline for in vivo studies. C57BL/6J mice were injected with B16-F10 parental or PBRM1 knockout cells (black arrow). After 3 days tumour growth and twice weekly after this, mice were treated with either vehicle or 50mg/kg BOS172722 and their tumour volume was measured (green arrows). **g** Combined Kaplan-Meier survival curve showing survival of mice with tumours derived from B16-F10 parental or PBRM1 knockout cells, treated with vehicle (VEH) or BOS172722 (BOS), corresponding to data from individual cell lines in Figure 7i-k. n=9 mice per condition, and survival was compared using the logrank (Mantel-Cox) test. For KO2 +VEH versus KO2 +BOS, **p=0.0016; for KO1 +VEH versus PAR +VEH, **p=0.0010; for KO2 +VEH versus PAR +VEH, *p=0.0445. **h** Graph showing tumour volumes over indicated times of mice injected with B16-F10 parental or PBRM1 knockout cells, treated with either vehicle (VEH) or BOS172722 (BOS). n=9 per condition, and graph shows data up until latest timepoint where all mice were still surviving. Tumour growth corresponds to area under the curve (A.U.C.) data from Figures 7l-n. Line indicates mean tumour volume ± SEM. Source data are provided as a source data file.

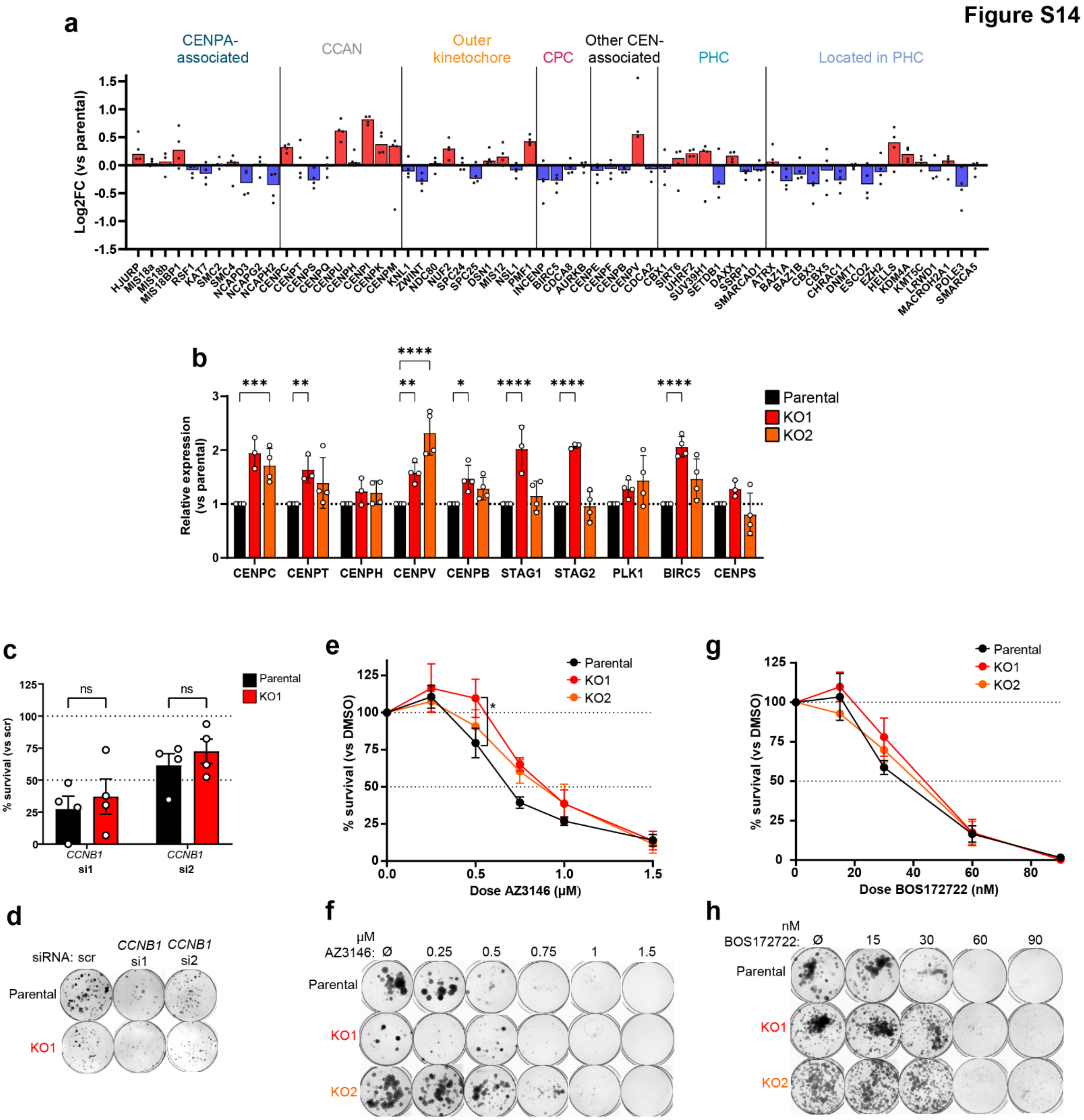

Supplementary Figure 14. PBRM1 KOs in 786-O do not exhibit centromere phenotypes. a Median Log2FC of annotated centromere and pericentromere proteins in RPE1 PBRM1 knockouts compared to parental cells. Points correspond to individual knockout clones from one of two independent biological replicates. **b** Expression levels of a panel of centromere-associated genes. RT-qPCR was used to compare transcript levels in four independent PBRM1 knockout clones in 786-O, relative to transcript levels in parental cells. *GAPDH* and *PPIA* were used for normalisation. Points correspond to independent biological replicates. *n*=4, mean±SEM, and data were analysed by 2way ANOVA with Dunnett's test, *****p*<0.0001. For CENPC condition, ****p*=0.0008; for CENPT, ***p*=0.0074; for CENPV, ***p*=0.0086; and for CENPB, **p*=0.0466. **c** Clonogenic survival of 786-O parental and PBRM1 knockouts after *CCNB1* RNAi with two independent *CCNB1* siRNAs, normalised to survival after treatment with a scramble (scr) siRNA. Points correspond to independent biological replicates, *n*=4, mean±SEM, and data were analysed by 2way ANOVA with Dunnett's test. **d** Representative image of colony formation from clonogenic survival assay, after treatment with the indicated siRNA as indicated in **c**. **e&g** Clonogenic survival of 786-O parental and PBRM1 knockout cells after treatment with increasing doses of the Mps1 inhibitors (e) AZ3146, and (g) BOS172722, compared to survival with treatment with DMSO alone (Ø). *n*=4, mean±SEM, and data were analysed by 2way ANOVA with Dunnett's test, **p*=0.0335. **f&h** Representative image of colony formation from clonogenic survival assays in **e** and **g**. Source data are provided as a source data file.

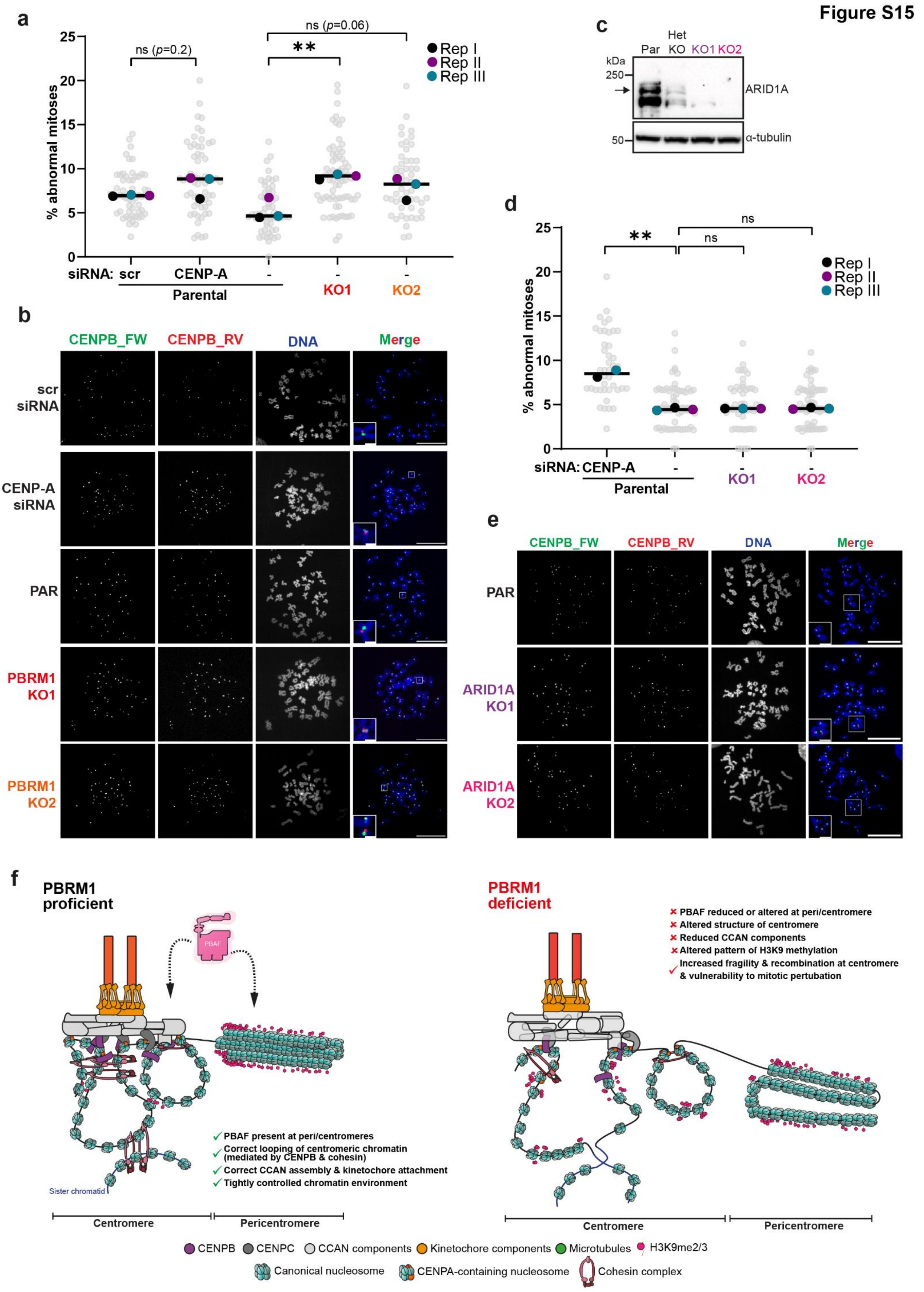

Figure S15
a
% abnormal mitoses
ns (p=0.2)
ns (p=0.06)
**
Rep I
Rep II
Rep III
siRNA: scr
CENP-A
Parental
KO1
KO2
b
CENPB_FW
CENPB_RV
DNA
Merge
scr siRNA
CENP-A siRNA
PAR
PBRM1 KO1
PBRM1 KO2
c
Par
Het KO
KO1
KO2
kDa
250
50
ARID1A
α-tubulin
d
% abnormal mitoses
**
ns
ns
Rep I
Rep II
Rep III
siRNA: CENP-A
Parental
KO1
KO2
e
CENPB_FW
CENPB_RV
DNA
Merge
PAR
ARID1A KO1
ARID1A KO2
f
PBRM1 proficient
PBAF
PBAF present at peri/centromeres
Correct looping of centromeric chromatin (mediated by CENPB & cohesin)
Correct CCAN assembly & kinetochore attachment
Tightly controlled chromatin environment
Sister chromatid
Centromere
Pericentromere
PBRM1 deficient
PBAF reduced or altered at peri/centromere
Altered structure of centromere
Reduced CCAN components
Altered pattern of H3K9 methylation
Increased fragility & recombination at centromere & vulnerability to mitotic pertubation
Centromere
Pericentromere
CENPB
CENPC
CCAN components
Kinetochore components
Microtubules
H3K9me2/3
Canonical nucleosome
CENPA-containing nucleosome
Cohesin complex

Supplementary Figure 15. Centromere instability is conserved in other PBRM1 KOs but is not observed in ARID1A KOs. a Quantification of chromatid exchanges at centromeres in 1BR3 parental or PBRM1 knockouts, or parental cells treated with CENP-A or scramble siRNAs, defined as the percentage of chromosomes with aberrant centromeres in each metaphase spread (% abnormal mitoses). n=3, grey points indicate individual metaphases, and coloured points represent the median of independent biological replicates, with line at the median of these replicates. Data were analysed using two-sided t-test, **p=0.0070. At least 50 metaphases were analysed per condition. **b** Representative metaphase spreads from Cen-CO-FISH experiments in (a), with DNA stained with DAPI (blue), and centromeres hybridised with FISH probes against CENPB-box sequences (green & red). Scale bars correspond to 20μm. White boxes correspond to zoomed inset images, where scale bars correspond to 5μm. **c** Western blot for ARID1A in RPE1 parental and ARID1A knockouts (KO1 and KO2). A third clone (Het KO) was found to have a heterozygous mutation in ARID1A alleles and was not used in this study. α-tubulin is used as loading control, black arrow indicates band corresponding to ARID1A. **d** Quantification of chromatid exchanges at centromeres in RPE1 parental or ARID1A knockouts, defined as the percentage of chromosomes with aberrant centromeres in each metaphase spread (% abnormal mitoses). In two replicates, RPE1 parental cells were also treated with CENP-A siRNAs to validate the detection of chromatid exchanges. n=3 for all conditions except CENP-A siRNA (n=2), grey points indicate individual metaphases, and coloured points represent the median of independent biological replicates, with line at the median of these replicates. At least 59 metaphases were analysed per condition (>40 in CENP-A condition), and data with 3 biological replicates were analysed using two-sided t-test. **e** Representative metaphase spreads from Cen-CO-FISH experiments in (d), with DNA stained with DAPI (blue), and centromeres hybridised with FISH probes against CENP-B-box sequences (green & red). Scale bars correspond to 20μm. White boxes correspond to zoomed inset images, where scale bars correspond to 2μm. **f** Model for how PBRM1 loss affect the centromeric and pericentromeric chromatin environment. Source data are provided as a source data file.

Table S1: oligonucleotides used in this study

DNA primers		
**Name**	**Sequence**	**Application**
PBRM1_sgRNA2_FW	**CACCG**TCAGCGGGGACTTTGATGAT	Generation of PBRM1 knockout
PBRM1_sgRNA2_RV	**AAAC**ATCATCAAAGTCCCCGCTGA**C**	Generation of PBRM1 knockout
SMARCA4_ex2_FW1.1	**ACCG**CTGGGCGGAACTCCTCGGCC	Generation of SMARCA4 knockout
SMARCA4_ex2_RV1.2	**AAAC**GGCCGAGGAGTTCCGCCCAG	Generation of SMARCA4 knockout
SMARCA4_ex2_FW1.3	**ACCG**TCTGGAGTGGACATCTTCAC	Generation of SMARCA4 knockout
SMARCA4_ex2_FW1.4	**AAAC**GTGAAGATGTCCACTCCAGA	Generation of SMARCA4 knockout
ARID1A_sg2_FW	**CACCG**ATGGTCATCGGGTACCGCTG	Generation of ARID1A knockout
ARID1A_sg2_RV	**AAAC**CAGCGGTACCCGATGACCATC	Generation of ARID1A knockout
CENPC_qPCR_FW	AGGAAGGCCATCAGGAGGAT	RT-qPCR
CENPC_qPCR_RV	GGGTCCTTTACCCTCGTTGG	RT-qPCR
CENPT_qPCR_FW	CCACGGAAGACTCAGAAACCA	RT-qPCR
CENPT_qPCR_RV	AGTCTTGGCTTCTTCATCATATTCT	RT-qPCR
CENPH_qPCR_FW	AGGCTGAGAGCACAGACAAAA	RT-qPCR
CENPH_qPCR_RV	GCAGTTGAAAGTCTCATCCTGTC	RT-qPCR
CENPV_qPCR_FW	GGGAGCTGAGCACATAACGA	RT-qPCR
CENPV_qPCR_RV	GGGGCAATTCCGAAGCCT	RT-qPCR
CENPB_qPCR_FW	ACCTAGATTGGCAGCACCAC	RT-qPCR
CENPB_qPCR_RV	GGCCAAATGGGGAGGAAAGA	RT-qPCR
STAG1_qPCR_FW	ATTGGCGTGTGGAAAATGCC	RT-qPCR
STAG1_qPCR_RV	CCTCAAAGGCAATCCTCAGC	RT-qPCR
STAG2_qPCR_FW	GACCGCCACTTTCAAAACCC	RT-qPCR
STAG2_qPCR_RV	GTGGCCACCTCCCCC	RT-qPCR
PLK1_qPCR_FW	CCGCAATTACATGAGCGAGC	RT-qPCR
PLK1_qPCR_RV	TGAGCTTGGTGTGATCCTGG	RT-qPCR
BIRC5_qPCR_FW	CCGCAATTACATGAGCGAGC	RT-qPCR
BIRC5_qPCR_RV	TGAGCTTGGTGTGATCCTGG	RT-qPCR
CENPS_qPCR_FW	CCAGGAGGAGTAATTCACTGCTAAA	RT-qPCR
CENPS_qPCR_RV	GCCATATGCATTTCTAGGTGGC	RT-qPCR
GAPDH_qPCR_FW	ATTCCACCCATGGCAAATTC	RT-qPCR
GAPDH_qPCR_RV	TCTCGCTCCTGGAAGATGGT	RT-qPCR
PPIA_qPCR_FW	AGGGTTCCTGCTTTCACAGA	RT-qPCR
PPIA_qPCR_RV	CTTGCCACCAGTGCCATTAT	RT-qPCR

Table S2: antibodies used in this study

Primary antibodies					
**Target**	**Source**	**Product**	**Host species**	**Application**	**Dilution**
PBRM1	Novus	NBP2-76400	Rabbit	WB	1:1000
				IF	1:100
	Active Motif	AB_279361	Rabbit	CUT&RUN	1:50
H3K9me3	Abcam	ab-176916	Rabbit	IF	1:2000
				CUT&RUN	1:50
NDC80	Abcam	ab3613	Mouse	IF	1:1000
CCNB1	Santa Cruz	sc-245	Mouse	WB	1:200
ARID1A	CST	12354	Rabbit	WB	1:1000
SMARCA4	Santa Cruz	sc-17796	Mouse	WB	1:2000
	Abcam	ab110641	Rabbit	CUT&RUN	1:25
H3K9me2	Millipore	05-1249	Mouse	CUT&RUN	1:50
CENP-B	Abcam	ab25734	Rabbit	CUT&RUN	1:50
				IF	1:500
CENP-A	Abcam	ab-13939	Mouse	WB	1:1000
	Invitrogen	MA1-20832	Mouse	IF	1:1000
IgG isotype control	CST	66362	Rabbit	CUT&RUN	1:20
α-tubulin	Abcam	ab-7291	Mouse	WB	1:10000
				IF	1:10000
β-actin-HRP	Sigma	A3854	Mouse	WB	1:40000

Secondary antibodies					
**Target**	**Source**	**Product code**	**Host species**	**Application**	**Dilution**
Anti-mouse HRP	Agilent Dako	P0260	Rabbit	WB	1:10000
Anti-rabbit HRP	Agilent Dako	P0448	Goat	WB	1:10000
Anti-mouse Alexa Fluor 488	Invitrogen	A-11029	Goat	IF	1:500
Anti-rabbit Alexa Fluor 488	Invitrogen	A-21206	Donkey	IF	1:500
Anti-mouse Alexa Fluor 555	Invitrogen	A-21422	Goat	IF	1:500
Anti-rabbit Alexa Fluor 555	Invitrogen	A-31572	Donkey	IF	1:500
Anti-mouse Alexa Fluor 647	Invitrogen	A-21235	Goat	IF	1:500
