# Supplementary material for: PBRM1 directs PBAF to pericentromeres and protects centromere integrity
Source: Nat Commun. 2025 Feb 26;16:1980. doi: 10.1038/s41467-025-57277-9 (PMC11865495; doi:10.1038/s41467-025-57277-9)
Supplement: Supplementary file 3 — Reporting Summary [file 41467_2025_57277_MOESM3_ESM.pdf]

nature portfolio

Corresponding author(s): Jessica A. Downs

Last updated by author(s): Jan 7, 2025

Reporting Summary

Nature Portfolio wishes to improve the reproducibility of the work that we publish. This form provides structure for consistency and transparency in reporting. For further information on Nature Portfolio policies, see our Editorial Policies and the Editorial Policy Checklist.

Statistics

For all statistical analyses, confirm that the following items are present in the figure legend, table legend, main text, or Methods section.

n/a	Confirmed	
☐	☒	The exact sample size (*n*) for each experimental group/condition, given as a discrete number and unit of measurement
☐	☒	A statement on whether measurements were taken from distinct samples or whether the same sample was measured repeatedly
☐	☒	The statistical test(s) used AND whether they are one- or two-sided<br>*Only common tests should be described solely by name; describe more complex techniques in the Methods section.*
☒	☐	A description of all covariates tested
☐	☒	A description of any assumptions or corrections, such as tests of normality and adjustment for multiple comparisons
☐	☒	A full description of the statistical parameters including central tendency (e.g. means) or other basic estimates (e.g. regression coefficient) AND variation (e.g. standard deviation) or associated estimates of uncertainty (e.g. confidence intervals)
☐	☒	For null hypothesis testing, the test statistic (e.g. *F*, *t*, *r*) with confidence intervals, effect sizes, degrees of freedom and *P* value noted<br>*Give P values as exact values whenever suitable.*
☒	☐	For Bayesian analysis, information on the choice of priors and Markov chain Monte Carlo settings
☒	☐	For hierarchical and complex designs, identification of the appropriate level for tests and full reporting of outcomes
☒	☐	Estimates of effect sizes (e.g. Cohen's *d*, Pearson's *r*), indicating how they were calculated

Our web collection on statistics for biologists contains articles on many of the points above.

Software and code

Policy information about availability of computer code

Data collection	Proteome Discoverer (v2.2 or 2.4), SequestHT, BD FACSDiva, SlideBook imaging software (3i), StepOne Plus (v2.3)(Applied Biosystems), Depmap (Expression Public 23Q2 and Proteomics)
Data analysis	FlowJo (v10.8.1), GSEA (v4.3.2), R (v4.2.1 or 4.2.2 or 4.2.3) and RStudio (v2021.09.0 or 2023.03.1), ComplexHeatmap (v2.20.00), TrimGalore (v0.6.6), Bowtie2 (v2.4.2), Sambamba (v0.5.0), Picard tools (v2.23.8), SAMtools (v1.11), BAMtools (v2.5.1), Deeptools (v3.1.3) , genefilter (v1.78.), ggVennDiagram (v1.5.2), FastQC (v0.11.9), Ribodetector (v0.2.7), RSeQC (v4.0.0), STAR (v2.7.6a), HTSeqCount (v0.12.4), DESeq2 (v1.38.3), bedtools (v2.29.2), ImageJ (v1.5.3 or 1.5.4), CellProfiler (v4.0.7), Adobe Illustrator (v27.5), BioRender, BBmap (v38.84), kmc (v3.2.1), dplyr (v1.1.0), Tidyverse (v2.0.0), Tibble (v3.2.0), GNU datamash (v1.1.0), MACS2 (v2.2.7.1), ChIPseeker (v1.40.0), intervene (v0.6.5), EnrichedHeatmap (v1.34.0), seqkit (v2.5.1), trimmomatic (v0.39), datamash (v1.1.0)

For manuscripts utilizing custom algorithms or software that are central to the research but not yet described in published literature, software must be made available to editors and reviewers. We strongly encourage code deposition in a community repository (e.g. GitHub). See the Nature Portfolio guidelines for submitting code & software for further information.

Data

Policy information about availability of data

All manuscripts must include a data availability statement. This statement should provide the following information, where applicable:

- Accession codes, unique identifiers, or web links for publicly available datasets
- A description of any restrictions on data availability
- For clinical datasets or third party data, please ensure that the statement adheres to our policy

Mass spectrometry source data generated in this study have been deposited to the ProteomeXchange Consortium via the PRIDE partner repository under the accession code PXD043209.

The total RNA-seq data reported here have been deposited in the GEO database under accession number GSE235342.

The CUT&RUN data generated in this study have been deposited under accession number GSE235294.

Research involving human participants, their data, or biological material

Policy information about studies with human participants or human data. See also policy information about sex, gender (identity/presentation), and sexual orientation and race, ethnicity and racism.

Reporting on sex and gender	n/a
Reporting on race, ethnicity, or other socially relevant groupings	n/a
Population characteristics	n/a
Recruitment	n/a
Ethics oversight	n/a

Note that full information on the approval of the study protocol must also be provided in the manuscript.

Field-specific reporting

Please select the one below that is the best fit for your research. If you are not sure, read the appropriate sections before making your selection.

☒ Life sciences ☐ Behavioural & social sciences ☐ Ecological, evolutionary & environmental sciences

For a reference copy of the document with all sections, see nature.com/documents/nr-reporting-summary-flat.pdf

Life sciences study design

All studies must disclose on these points even when the disclosure is negative.

Sample size	Sample sizes were decided based on standard practise from the field, and experiments generally had n=3 or n=4 biological replicates. Multiple knockout clones were used to confirm results. Three technical replicates were also used where appropriate, i.e. for survival assays, RT-qPCR, proliferation assays, etc.
Data exclusions	Outliers were removed using Grubbs' test, and only data with a significance level of 0.05 were excluded. A single proteomics biological replicate in 1BR3-hTERT cells was also excluded as it was identified as an outlier based on principal component analysis (PCA).
Replication	Biological replicates were used to ensure data was replicable. Ranges of doses of drugs and timepoints were also used, as well as multiple assays to confirm results. Multiple knockout clones were also used to ensure observations were replicated between cells. Proteomic and transcriptomic data were also used to confirm results.
Randomization	Randomisation was not used in this paper - rather results were compared to control samples.
Blinding	Blinding was used for manual analyses of clonogenic survival assays and Cen-CO-FISH quantification.

Reporting for specific materials, systems and methods

We require information from authors about some types of materials, experimental systems and methods used in many studies. Here, indicate whether each material, system or method listed is relevant to your study. If you are not sure if a list item applies to your research, read the appropriate section before selecting a response.

Materials & experimental systems

n/a	Involved in the study
☐	☒ Antibodies
☐	☒ Eukaryotic cell lines
☒	☐ Palaeontology and archaeology
☐	☒ Animals and other organisms
☒	☐ Clinical data
☒	☐ Dual use research of concern
☒	☐ Plants

Methods

n/a	Involved in the study
☒	☐ ChIP-seq
☐	☒ Flow cytometry
☒	☐ MRI-based neuroimaging

Antibodies

Antibodies used

PBRM1 (Novus NBP2- 76400, Active Motif AB_2793612), CCNB1 (Santa Cruz sc-245), α-tubulin (Abcam ab7291), β-actin-HRP (Sigma A3854), CENPA (Abcam ab13939, Invitrogen MA1-20832), SMARCA4 (Santa Cruz sc-17796, Abcam ab110641), ARID1A (CST 12354), CENPB (Abcam ab25734), H3K27me3 (CST 9733), IgG isotype control (CST 66362), H3K9me2 (Millipore 05-1249), H3K9me3 (Abcam ab176916), NDC80 (Abcam, ab3613)
Anti-mouse HRP (Agilent Dako P0260), Anti-rabbit HRP (Agilent Dako P0448), Anti-mouse Alexa Fluor 488 (Invitrogen A-11029), Anti-rabbit Alexa Fluor 488 (Invitrogen A-21206), Anti-mouse Alexa Fluor 555 (Invitrogen, A-21422), Anti-mouse Alexa Fluor 647 (Invitrogen A-21235), Anti-rabbit Alexa Fluor 555 (Invitrogen, A-31572)

Validation

As the following antibodies used in this study are commercially available, links for validation on the manufacturer's website are provided below, as well as details of figures where validation was performed in the lab with siRNA or knockouts against the target gene. PBRM1 (Novus NBP2- 76400: https://www.novusbio.com/products/baf180-pb1-antibody-bl-39-2c3_nbp2-76400?srsltid=AfmBOore2M7nKNqMBhsY8KzLtCBWQcb5P6A2XUAplVCuukAC4rkhxPgM, Fig. 1b, Fig. 7d, Supplementary Fig. 1a-d; Active Motif AB_2793612: https://www.activemotif.com/catalog/details/61381/pbrm1-antibody-pab), CCNB1 (Santa Cruz sc-245: https://www.scbt.com/p/cyclin-b1-antibody-gns1?srsltid=AfmBOorM0p_YSHgmqpVv5udF9_5fY-5RgmxFmrmhGnQp0x27U5bq7MkZ, Supplementary Fig. 12c,e), α-tubulin (Abcam ab7291: https://www.abcam.com/en-us/products/primary-antibodies/alpha-tubulin-antibody-dm1a-loading-control-ab7291?srsltid=AfmBOopGqsQGNq0mz0Ej65RbpJewd3zLErj7AzR3py_9WzTMOfbriAu9), β-actin-HRP (Sigma A3854: https://www.sigmaaldrich.com/GB/en/product/sigma/a3854), CENPA (Abcam ab13939: https://www.citeab.com/antibodies/722546-ab13939-anti-cenpa-antibody-3-19-chip-grade; Fig. 8g; Invitrogen MA1-20832: https://www.thermofisher.com/antibody/product/CENPA-Antibody-clone-3-19-Monoclonal/MA1-20832), SMARCA4 (Santa Cruz sc-17796: https://www.scbt.com/p/brg-1-antibody-g-7?srsltid=AfmBOor9L4ZlWzHl9nUyrqaLLb9ATGSXT3sKq7SFB9v5EkR2B0bMb8zd, Supplementary Fig. 7b; Abcam ab110641: https://www.abcam.com/en-us/products/primary-antibodies/brg1-antibody-epncir111a-ab110641?srsltid=AfmBOopAeiVsnJT6HEaFD6wabOeyfhx0o97MU2c4o2tSolWIgqrpa71l), ARID1A (CST 12354: https://www.cellsignal.com/products/primary-antibodies/arid1a-baf250a-d2a8u-rabbit-mab/12354?srsltid=AfmBOooTEDIN9HsjvPSAOWaKkqoqSAzmm4r1vJsEXsvcZ1sDXBPSnSR-, Supplementary Fig. 15c), CENPB (Abcam ab25734: https://www.abcam.com/en-us/products/primary-antibodies/cenpb-antibody-ab25734?srsltid=AfmBOooT0pZRuaZXnG7-17x_fpvfdx718cy0tRDGjBynq1D0Thj5ptwF), H3K27me3 (CST 9733: https://www.cellsignal.com/products/primary-antibodies/tri-methyl-histone-h3-lys27-c36b11-rabbit-mab/9733?srsltid=AfmBOoqbOYJnUfiQO8d9RSXOcDSeEWG0GMamAB06FFieWfT5Uf6QFCx6), IgG isotype control (CST 66362: https://www.cellsignal.com/products/primary-antibodies/rabbit-da1e-mab-igg-xp-isotype-control-cut-amp-run/66362?srsltid=AfmBOor9AxDTImmp3Dp54u0RDXsYvbR_Taa1CJByTHa_OkwPKUwW05S-), H3K9me2 (Millipore 05-1249: https://www.merckmillipore.com/GB/en/product/Anti-Dimethyl-Histone-H3-Lys9-Antibody-clone-CMA307,MM_NF-05-1249), H3K9me3 (Abcam ab176916: https://www.abcam.com/en-us/products/primary-antibodies/histone-h3-tri-methyl-k9-antibody-epr16601-chip-grade-ab176916?srsltid=AfmBOooHXsC7Q4SddqVpUR0kGY0RHVZzaWDMEJU7X4X10QAM4j5T3V8b), NDC80 (Abcam, ab3613: https://www.abcam.com/en-us/products/primary-antibodies/hec1-hec-antibody-9g3-ab3613?srsltid=AfmBOorGu_ABC2eHfDzCsvUDbQBDS4BUoo2I4fc5j8_2QcZm-EVnEr2t)
Anti-mouse HRP (Agilent Dako P0260: https://www.agilent.com/store/en_US/Prod-P026002-2/P026002-2), Anti-rabbit HRP (Agilent Dako P0448: https://www.agilent.com/store/productDetail.jsp?catalogId=P044801-2), Anti-mouse Alexa Fluor 488 (Invitrogen A-11029: https://www.thermofisher.com/antibody/product/Goat-anti-Mouse-IgG-H-L-Highly-Cross-Adsorbed-Secondary-Antibody-Polyclonal/A-11029), Anti-rabbit Alexa Fluor 488 (Invitrogen A-21206: https://www.thermofisher.com/antibody/product/Donkey-anti-Rabbit-IgG-H-L-Highly-Cross-Adsorbed-Secondary-Antibody-Polyclonal/A-21206), Anti-mouse Alexa Fluor 555 (Invitrogen, A-21422: https://www.thermofisher.com/antibody/product/Goat-anti-Mouse-IgG-H-L-Cross-Adsorbed-Secondary-Antibody-Polyclonal/A-21422), Anti-mouse Alexa Fluor 647 (Invitrogen A-21235: https://www.thermofisher.com/antibody/product/Goat-anti-Mouse-IgG-H-L-Cross-Adsorbed-Secondary-Antibody-Polyclonal/A-21235), Anti-rabbit Alexa Fluor 555 (Invitrogen, A-31572: https://www.thermofisher.com/antibody/product/Donkey-anti-Rabbit-IgG-H-L-Highly-Cross-Adsorbed-Secondary-Antibody-Polyclonal/A-31572)

Eukaryotic cell lines

Policy information about cell lines and Sex and Gender in Research

Cell line source(s)

1BR3-hTERT cells were a gift from Professor Penny Jeggo, Sussex University.
The following cells were obtained from ATCC, with catalogue numbers indicated in brackets: hTERT-RPE1 (CRL-4000), U2OS (HTB-96), B16-F10 (CRL-6475), 786-O (CRL-1932), 769-P (CRL-1933), Caki-1 (HTB-46), and Caki-2 (HTB-47).
HEK293TN were from Systems Biosciences, (catalogue no LV900A-1)
RCC4-VO were obtained from ECACC (catalogue no. 03112702). RCC-FG2 were obtained from CLS (catalogue no. 300249).

Authentication	1BR3-hTERT cells were generated and authenticated by Professor Penny Jeggo. All other cell lines were all obtained commercially with authentication.
Mycoplasma contamination	Cell lines were tested regularly (every 2 months) for mycoplasma contamination and were negative.
Commonly misidentified lines (See ICLAC register)	No commonly misidentified lines were used in this study.

Animals and other research organisms

Policy information about studies involving animals; ARRIVE guidelines recommended for reporting animal research, and Sex and Gender in Research

Laboratory animals	C57/BL6J mice were used at 6-8 weeks of age
Wild animals	This study did not involve wild animals
Reporting on sex	Only female mice were used in this study.
Field-collected samples	This study did not involve samples collected from the field.
Ethics oversight	All animal work was carried out under UK Home Office Project Licence P5541E04C (Establishment Licence X702B0E74) granted under the Animals (Scientific Procedures) Act 1986 and approved by the 'Animal Welfare and Ethical Review Body' at the Institute of Cancer Research (ICR).Protocol number BSU_SPF_1467.

Note that full information on the approval of the study protocol must also be provided in the manuscript.

Flow Cytometry

Plots

Confirm that:

☐ The axis labels state the marker and fluorochrome used (e.g. CD4-FITC).

☐ The axis scales are clearly visible. Include numbers along axes only for bottom left plot of group (a 'group' is an analysis of identical markers).

☒ All plots are contour plots with outliers or pseudocolor plots.

☐ A numerical value for number of cells or percentage (with statistics) is provided.

Methodology

Sample preparation	For FACS analyses, cells were fixed with 70% ethanol at -20oC overnight. For single cell sorting, live cells were resuspended in sorting media (PBS containing 1mM EDTA, 25mM HEPES (pH=7.0), and 1% FBS).
Instrument	BD LSR II<br>BD FACSymphony A5<br>BD FACSAria III sorter
Software	BD FACSDiva<br>FlowJo v.10.8.1
Cell population abundance	At least 10,000 single cells were detected before gating.
Gating strategy	Cells were first identified using FSC-A vs SSC-A to gate out debris. Single cells were then identified using FSC-A vs FSC-H. Cells

☒ Tick this box to confirm that a figure exemplifying the gating strategy is provided in the Supplementary Information.
